# Genome-wide expression analysis suggests a crucial role of dysregulation of matrix metalloproteinases pathway in undifferentiated thyroid carcinoma

**DOI:** 10.1186/s12864-015-1372-0

**Published:** 2015-03-18

**Authors:** Jesús Espinal-Enríquez, Said Muñoz-Montero, Ivan Imaz-Rosshandler, Aldo Huerta-Verde, Carmen Mejía, Enrique Hernández-Lemus

**Affiliations:** National Institute of Genomic Medicine, Periférico Sur 4809, Arenal Tepepan, TlalpanMéxico City, 14610 México; Center for Sciences of Complexity (C3), UNAM, Ciudad Universitaria, México City, 01010 México; Faculty of Natural Sciences, Autonomous University of Querétaro, P.O. Box 184, Querétaro, 76230 México

**Keywords:** Anaplastic thyroid cancer, Inhibition of matrix metalloproteinases, Pathway analysis, Thyroid carcinoma progression, Genome-wide analysis

## Abstract

**Background:**

Thyroid cancer (TC) is the most common malignant cancer of the Endocrine System. Histologically, there are three main subtypes of TC: follicular, papillary and anaplastic. Diagnosing a thyroid tumor subtype with a high level of accuracy and confidence is still a difficult task because genetic, molecular and cellular mechanisms underlying the transition from differentiated to undifferentiated thyroid tumors are not well understood.

A genome-wide analysis of these three subtypes of thyroid carcinoma was carried out in order to identify significant differences in expression levels as well as enriched pathways for non-shared molecular and cellular features between subtypes.

**Results:**

Inhibition of matrix metalloproteinases pathway is a major event involved in thyroid cancer progression and its dysregulation may result crucial for invasiveness, migration and metastasis. This pathway is drastically altered in ATC while in FTC and PTC, the most important pathways are related to DNA-repair activation or cell to cell signaling events.

**Conclusion:**

A progression from FTC to PTC and then to ATC was detected and validated on two independent datasets. Moreover, PTX3, COLEC12 and PDGFRA genes were found as possible candidates for biomarkers of ATC while GPR110 could be tested to distinguish PTC over other tumor subtypes. The genome-wide analysis emphasizes the preponderance of pathway-dysregulation mechanisms over simple gene-malfunction as the main mechanism involved in the development of a cancer phenotype.

**Electronic supplementary material:**

The online version of this article (doi:10.1186/s12864-015-1372-0) contains supplementary material, which is available to authorized users.

## Background

Thyroid cancer (TC) is the most common malignant tumor of the endocrine system. TC is currently presenting an unexplained incidence. Although it can occur at any age, onset is more common after 30 y.o.; its aggressiveness significantly increasing in elderly patients. TC does not always cause symptoms being its first sign a thyroid nodule.

Thyroid neoplasms are histologically classified into follicular adenoma (FA), follicular thyroid cancer (FTC), papillary thyroid cancer (PTC) and anaplastic thyroid cancer (ATC). FTC and PTC are differentiated tumors with low risk of recurrence and good prognosis. On the other hand, ATC is an undifferentiated thyroid cancer, usually diagnosed at an advanced stage and thus not responding to chemotherapy; therefore frequently leading to a fatal prognosis [1]. Prognosis is related to stage at diagnosis and 5-year survival is 99% for patients with localized disease versus 58% for patients with distant metastases. Almost all differentiated thyroid carcinomas can be suppressed either by surgery, replacement therapy through thyroid stimulating hormone (TSH) or iodine-131 treatment. To date, physicians continue to face two major problems when trying to determine the best management strategy for patients with thyroid nodules. The first one is to determine the type of ailment. In fact, 15-30 % of thyroid nodules evaluated by fine-needle aspiration cytology are not clearly benign or malignant [2]. A second dilemma is the identification of the most aggressive cases, mainly papillary and anaplastic thyroid tumors. These could be treated undergoing relentless therapy and follow-up instead of invasive and costly procedures [3].

Thyroid tumors result from the accumulation of a variety of genetic and epigenetic alterations that lead to gain-of-function in oncogenes and loss-of-function in tumor suppressor genes. An understanding of the different events involved in gene pathways would advance knowledge of thyroid cancer and therefore, improve the strategic course of treatment since to this date a reliable molecular test to depict differences in adenomatoid nodule, adenoma, and carcinoma has not been developed [4,5].

*Follicular Carcinoma.* It accounts for 5-15 *%* of thyroid carcinomas overall. Mutations of the RAS proto-oncogene have been linked to follicular thyroid carcinoma in up to 80 *%* follicular tumors [3]. The presence of the RAS mutation does not differentiate follicular carcinoma from follicular adenoma but is thought to play an important role in the transformation of follicular cells into carcinoma [6]. Moreover, PAX8 is a transcription factor that is essential for normal thyroid gland development when fusion protein with PPAR- *γ* has shown to be oncogenic [7]. PTEN gene activation of the PI3K/AKT signaling pathway is thought to promote the development of follicular and anaplastic thyroid carcinomas, as well as breast cancer [8].

*Papillary Carcinoma.* PTC is a primary thyroid cancer of follicular epithelial cell origin with distinctive nuclear features. PTC is the most common subtype of thyroid cancer, comprising 80 *%* of all thyroid carcinomas [9,10]. It is not possible to predict which tumors will behave more aggressively; however, various staging systems provide parameters which can be used to determine a patient’s prognosis. In PTC, aberrant methylation of tumor suppressor genes such as TIMP3 (tissue inhibitor of metalloproteinase-3) and DAPK (death-associated protein kinase) has been associated with tumor aggressiveness [11]. Gene expression analysis showed that cyclin D1 (CCND1), is overexpressed in PTC, which is not detectable in normal thyroid tissue [12]. Most mutations found in papillary thyroid carcinoma involve the common signaling pathway involving RET/PTC-RAS-BRAF. The biological effects of this pathway include changes in the cytoskeleton, cell proliferation, and differentiation [13].

*Anaplastic (Undifferentiated) Thyroid Carcinoma.* In stark contrast to other thyroid carcinomas, undifferentiated thyroid carcinoma is one of the most aggressive malignancies known [14]. It accounts for <5- 14*%* of thyroid malignancies. ATC is a tumor composed of undifferentiated tumor cells with immunohistochemical or ultrastructural evidence of epithelial derivation. In some instances, it is present in association with a differentiated carcinoma [2]. Most of these cases of undifferentiated carcinoma arise predominantly in association with PTC and other poorly differentiated carcinomas [14]. ATC (about 90 %) occur in the background of differentiated thyroid carcinoma (DTC), suggesting that DTC is a precursor agent [2]. This accumulation of chromosomal abnormalities along with ensuing gene dysregulation lead to loss of cell cycle control, signal transduction activation, and it is likely the underlying reason for its aggressive clinical behavior.

Multiple pathways of genetic alterations could lead to development of ATC, as not all ATC have identical genetic profiles. Most common genetic abnormalities in ATC involve RET, p53, RAS, BRAF, and *β*-catenin genes [2]. PIK3CA gene mutations have been described in 23 *%* of ATC [15]. This suggests a pathogenic role of the PI3K/AKT pathway in the transformation of PTC to ATC. The RAS-RAF-MEK-ERK-MAP kinase pathway is a signal transduction pathway that regulates cell proliferation and has also been associated with undifferentiated thyroid carcinomas [16]. BRAF mutations have been found in 29-70 *%* of PTCs and are associated with oncocytic variants of papillary carcinoma [2,5]. RAS genes are GTPases related to signal transduction for cell proliferation in the RAS-RAF-MEK-ERK-MAP kinase pathway. RAS activating point mutations are found in less than 10 *%* of PTCs [2,17]. However, RAS has been appeared more in the follicular variant of papillary carcinomas [2].

### Scope

Diagnosing the subtype of thyroid carcinoma with a high level of accuracy and confidence is still a difficult task. A better understanding of the genetic, molecular and cellular mechanisms underlying the transition from differentiated to undifferentiated thyroid tumor, as well as their progression is necessary to develop more specific and non-invasive treatments depending on the subtype of carcinoma. This is the rationale behind the present study. Here, by means of a Systems Biology approach, a genome-wide analysis of 11 anaplastic (ATC), 12 follicular (FTC) and 72 papillary thyroid carcinomas (PTC) samples, as well as 64 normal thyroid samples was carried out to understand the genetic and biochemical differences and similarities among them. Through pathway analysis, deregulation of genes involved in the inhibition of matrix metalloproteinases pathway has been pointed out as crucial for invasiveness, migration and metastasis.

The genetic analysis performed also suggests a progression from FTC to PTC and then to ATC, since a significant change in the expression level of some genes involved in determined pathways can be observed, mainly related to arrest of cell cycle, apoptosis, cell to cell signaling and mitosis. Furthermore, we established a possible crosstalk in cell-death-and-survival events that could be involved in the transition from PTC to ATC. Finally, a series of 4 genes that could be useful to determine the cancer subtype was set up. PTX3, COLEC12 and PDGFRA proved to differentiate anaplastic thyroid carcinomas from other subtype while GPR110 seems suitable to become a biomarker for papillary thyroid carcinomas. This analysis can be applied to other carcinogenic ailments as an alternative tool to discriminate among different tumor subtypes.

## Methods

**Figure 1:**
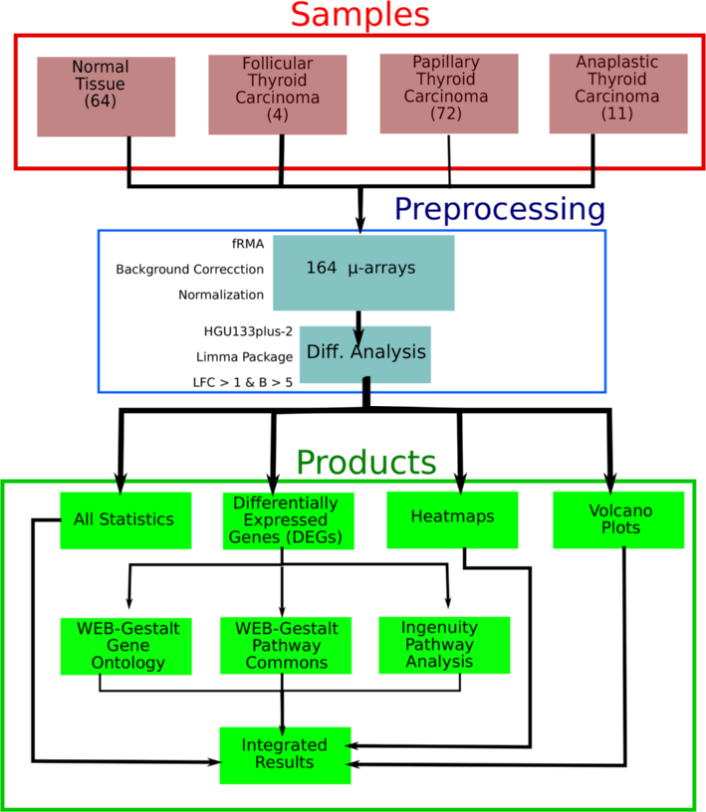
**Pipeline of the followed methodology to perform this study.** Red square represents the differences of the used samples. Blue square points to the preprocessing of the data. Finally, the green square is depicted for the results of the analysis.

A brief flowchart of the methods and samples used in this study is presented in Figure 1.

### Samples and pre-processing

Whole genome microarray gene expression data for 164 fine-needle extracted samples of thyroid tumors and normal thyroid tissue was obtain from publicly available archive databases (Accession keys: GSE3467, GSE33630, GSE3678, GSE5315). 64 belonged to healthy patients, 12 to FTC, 72 to PTC and 11 to ATC. 159 of those 164 samples proved viable to carry out the study in well-characterized groups (after data quality control and pre-processing). The actual sample was set up as follows:

- 64 normal thyroid (NT)
- 12 follicular thyroid carcinoma (FTC)
- 72 papillary thyroid carcinoma (PTC)
- 11 anaplastic thyroid carcinoma (ATC)

All samples were processed with the HG-U133_Plus_2-Affymetrix Human Genome U133 Plus 2.0 Array. (Supplementary Material 1). For microarray pre-processing and differential analysis, we used the Gene Expression Analysis Tool developed by Abraxas Biosystems ®; implementing a frozen Robust Multiarray Analysis (fRMA) -Background correction and normalization procedure [18]. Annotation was performed with the package hgu133plus2.db. Finally, limma, an [R] library for modeling gene expression data was used for differential expression analysis [19], considering as threshold the absolute value of the log-fold change (*LFC*)>1 and as significance measure the log-odds statistic (B) *B*>5 [20]. It is worth to mention that no experiments were performed on human tissue in this study; all the work presented here is analysis of previously published gene expression data.

### Gene expression analysis

In order to find Differentially Expressed Genes (DEGs) for average expression levels of the three different subtypes of carcinoma against normal controls, each subtype was compared. Once the overexpressed and underexpressed genes were established, a Gene Ontology (GO) Enrichment Analysis was performed in order to find those pathways enriched in each carcinoma and observe the shared gene sets for the three tumor subtypes. Significantly overexpressed genes were coded as **+**, whereas underexpressed molecules were coded as **-**. The gene ontology categories of those shared genes were then analyzed to search for common pathways or mechanisms that could present crosstalk between subtypes.

### Pathway analysis

Gene expression analysis (GEA) has become an extremely used technique to study the coordinated behavior of a large number of concomitant biological processes at the molecular level, aiming to develop a systemic wide phenomenological organismal characterization. However, GEA alone is often insufficient to gain biological insight as one relies on a very large number of differentially expressed genes commonly displaying disparate behavior among samples. To further increase our understanding and predictive capacities derived from such high-throughput experiments, a number of methods commonly termed *Pathway Analysis* have been developed in recent years [21-27]. This approach has been espacially fit to study complex phenotypes such as those associated with cancer [28]. Such complex cellular states are usually the consequence of a number of system-wide deregulation events rather than abnormalities at the single molecule level. Thus gene-centered studies are rather incomplete and often misguiding. Pathway analysis methods rely on strong probabilistic and computational methods to perform system-level analytics in carefully curated information repositories. Such repositories may be structured *Knowledge-based* resources, as well as unstructured *data- and literature- mining* search and retrieval applications.

Currently most of the existing pathway analysis methods are focused in the size of the sets of differentially expressed genes belonging to a given biological pathway. These methods are usually referred to as *enrichment methods* (EM). Other alternative pathway analysis techniques are based on the observation of the correlation between lists of genes belonging to certain pathways and their associated particular phenotypes (i.e. subsample sets), an approach known as *functional class scoring* (FCS). Both EM and FCS have proved to be quite useful yet still limited in their scope [21]. A number of advanced alternatives have been suggested recently. In reference [21], the authors combined a type of EM with perturbation analysis assessed by non-parametrical bootstrap procedures leading to the calculation of a global pathway significance statistic. Further improvement on this approach could be attained by resorting to probabilistic graphical methods to infer sample-specific pathway activities. This reasoning together with an integrative view was implemented by Vaske, et al. in a *factor-graph* based algorithm called PARADIGM [22]. To advance more on a mechanistic interpretation of *pathway activation*, a new context-specific metric termed a *Pathway Deregulation Score* (PDS) has been set forward [23]. PDS estimates the extent to which the behavior of a certain pathway in one sample deviates from that of a set of control samples. Once genome-wide PDS estimates are known, the *Pathifier* algorithm mines them to search for sample-specific pathway deregulation profiles and then statistical enrichment analysis is performed [23]. The *Pathifier* algorithm was recently supplemented with Cox regression and a regularized form of least-squares regression (the *L*^1^-LASSO -or *L*^1^-norm least absolute shrinkage and selection operator method) to achieve more accurate prognosis prediction.

Up to now, important computational and probabilistic ways to improve quantitative analysis on the available biological information databases and sources have been mentioned to underline their relevance. However, another important avenue of improvement most certainly relies on actual database build up. Biological information resources –both at the functional and molecular levels– may be either unstructured (large scale experimental data repositories, research papers and paper-collections as well as in-process annotations) or structured at different levels, from curated annotations and systematized repositories to quite specific and highly-curated *knowledge-based* resources.

In some sense, both approaches seek to develop better probabilistic and computational methods for pathway data analysis to gain access to fine knowledge-based sources of information. Hence, both are complementary. A nice example of the combination of powerful quantitative methods applied to carefully curated knowledge databases can be found in the recent paper by Verhaeg, et al. [25]. In it, the authors rely on probabilistic network Bayesian models for pathway activity inference applied to a manually curated database for specific pathways (the Wnt and ER pathways). Bayesian modeling was performed by using a clever modeling approach that considers three elements in a signaling pathway: a set of transcription complexes, a set fo target genes and a set of transcripts (from microarray gene expression experiments) corresponding to the target genes. The authors circumvent the common assumption of using gene expression intensity alone to model protein activity within a given pathway.

Though clearly relevant, computationally accurate methods based in unstructured databases require a very large number of experimental samples and eat up large computational capabilities, since most of these accurate methods rely in some form of higher statistical inference such as machine learning or Bayesian approaches. However, due to limitations in sample availability (precluding the study datasets with counts in the hundreds-to-thousands of samples), a two-way approach to pathway analysis was followed based on mining both large scale databases and highly curated knowledge bases –with all interactions taken from actual experimental data – implemented within the *causal network* philosophy [29,30].

Next, the Web-GESTALT enrichment tool was applied to perform a pathway analysis to uncover enriched pathways according to the set of DEGs found for each carcinoma subtype. Web-GESTALT is a quite comprehensive large scale data-mining tool. It consists of 78,612 functional categories across a dozen of large scale centrally and public databases. It performs EM and calculates statistical significance by means of FDR-corrected hypergeometric tests. We focused on large-scale commonalities and differences among the enriched pathways, using a false discovery rate (FDR) threshold = 0.01 and 2 genes as the minimum, to allow the set of genes to belong to a group. This procedure was applied as a broad, preliminary screen to filter-out pathway information.

Causal networks (CNs) were then generated through the *Ingenuity Pathway Analysis* methodology (IPA ®;, QIAGEN Redwood City www.qiagen.com/ingenuity). The approach taken to generate such CNs relied heavily on a highly curated knowledge-based source known as the *Ingenuity Knowledge Base* (IKB). IKB contains about 40,000 nodes representing mammalian genes and their products (transcripts, proteins, miRNAs, second messengers, etc.) as well as exogenous and endogenous chemical compounds. To date IKB reports more than 1,480,000 interactions between molecules. Such interactions are links between the nodes. These links represent *experimentally observed* cause-effect relationships relating to transcription, expression, activation, molecular modification, binding events and transport processes. Since these interactions have been experimentally measured they can be associated with a definite direction of the causal effect, either activation or inhibition of the above mentioned processes at a whole genome network-wide level. Consequently, then information sources are highly-curated since inference procedures are able to resort to two independent aspects. On one hand, enrichment scores are determined by hypergeometric tests or Fisher exact tests –depending on the statistical dependency conditions on the variables under consideration– that measure the overlap between observed and predicted gene sets. On the other, Z-score analysis asseses the match between observed and predicted up/down regulation patterns allowing for Bayesian scoring of the results [27].

To elaborate, within the IPA/IKB approach a *master* network is a directed multigraph *G*(*V*,*E*) constituted by nodes *v*∈*V* which are mammalian genes, proteins, miRNAS, second messengers or other molecules of interest ($\mathcal {C}(V) \simeq 40,000$ with  the cardinality or size of the set), whereas links *e*∈*E* are edges representing experimentally measured causal relations ($\mathcal {C}(E) \geq 1,480,000$). Since there can be more than one edge between two given nodes –for instance transcriptional activation and phosphorilation of one molecule, say A, may be *caused* by a single molecule B (albeit in a different state)–, the master network *G* is a multigraph. Given that the interactions may be either weighted or unweighted (depending on experimental availability of reliable quantitative data), *G* is in general a weighted, directed multigraph. For further methodological details please look up for reference [27].

This causal master network proved to be invaluable as a tool to find the common pathways in specific categories of interest such as cell death and survival, as well as cell to cell signaling processes for carcinoma subtype compared with the controls, since these categories were considered relevant in carcinoma progression. Finally, enriched pathways of each cancer subtype was contrasted and analyzed to point out significant differences among the three subtypes as well so to establish possible mechanisms of progression and malignancy.

## Results

### Differentially expressed genes for each carcinoma subtype

**Figure 2:**
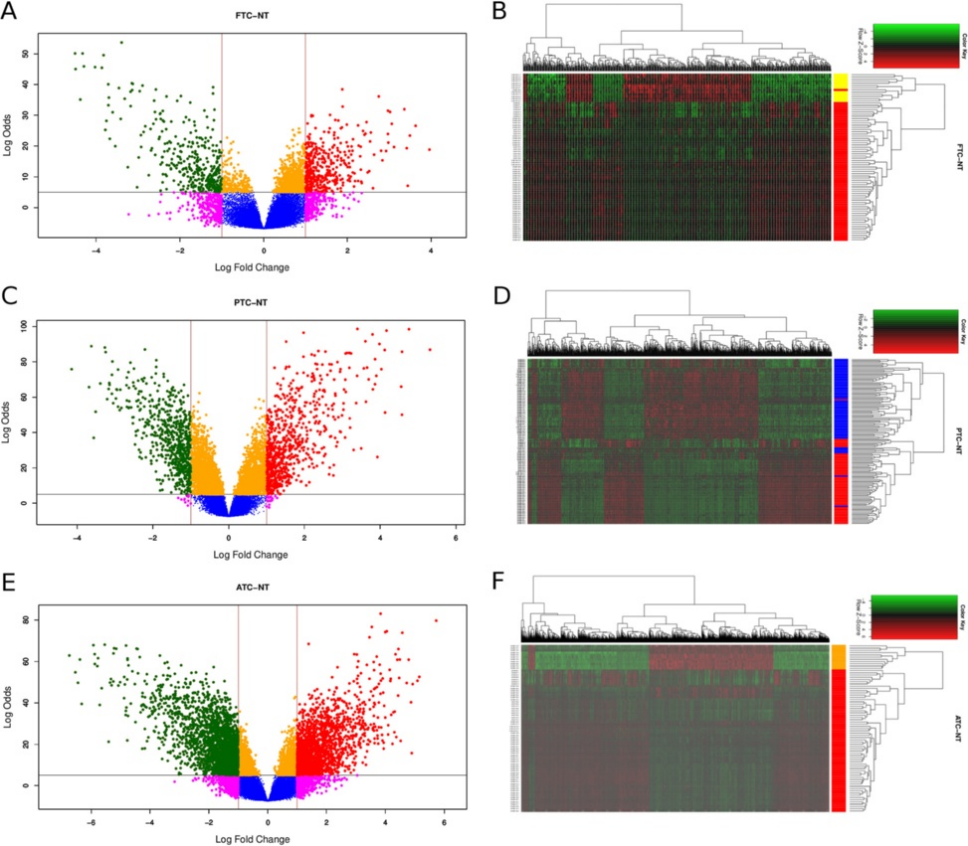
**Volcano plots and heatmaps of the DEGs for each subtype of carcinoma.** **A)** and **B)** represents the results for FTC, **C)** and **D)** for PTC and finally, **E)** and **F)** shows the results of ATC. It is worth to mention that for the case of ATC, the Red and green dots are more that those pink, blue and yellow. That is not the case for FTC or PTC. A volcano plot displays information about (the log-odds). Red dots are statistically significant changes in gene expression for whole genome experiments over-expressed genes while green dots are statistically based in calculations for the size of differential expression significant under-regulated genes (the log-fold change) and its statistical significance.

Results for differentially expressed genes for all the contrasts under consideration may be found in Table 1, Figure 2 and Additional file 1.

**Table 1:** **Differentially expressed genes for FTC, PTC and ATC**

**Type**	**Total DEGs**	***↑***	***↓***	**Top 5** ***↑***	**Top 5** ***↓***
				ZCCHC12 TENM1	DPT CHRDL1
FTC	579	289	290	CA12	CCL21
				ARHGAP36 PRR15	DCN ADH1B
				GABRB2 HMGA2	TPO DIO1
PTC	960	503	457	PRR15	ADH1B
				CHI3L1 ZCCHC12	PKHD1L1 TFF3
				POSTN MMP1	DIO1 TSHR
ATC	3220	1303	1917	VCAN	TG
				SPP1 TFPI2	SLC26A7 TPO

Differentially Expressed Genes (DEGs) for each subtype of carcinoma. It is also shown the top-5 overexpressed and underexpressed genes.

**Figure 3:**
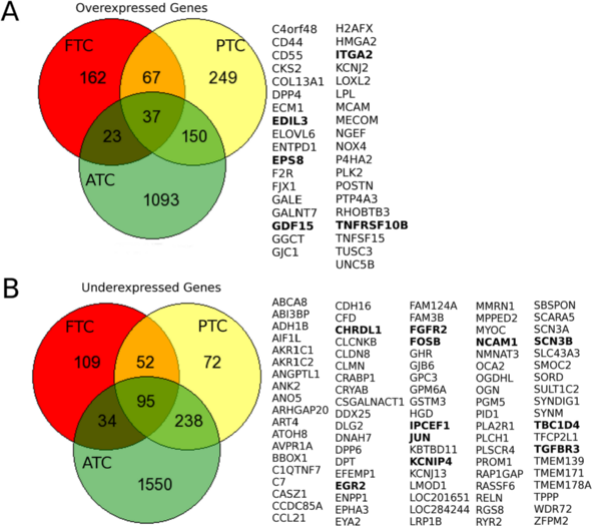
**Venn diagrams for the number of DEGs.** Both for overexpressed **(A)** and underexpressed **(B)** genes. For both cases, the Red circle contains FTC genes, yellow circles are for PTC and the green ones present ATC genes. The columns of the right show the shared genes for the three cancer subtypes.

*Overexpressed Genes.* Comparisons between FTC, PTC and ATC against the control show a subset of 37 common upregulated genes (Figure 3A). Other interesting results are that from 289 upregulated genes found in FTC samples, 162 were not shared by PTC or ATC. In the case of ATC only 8*%* of the upregulated genes are shared by PTC or FTC. Finally, in the case of PTC, approximately 40 *%* of the genes are shared by either ATC or FTC (Figure 3A).

*Underexpressed Genes.* In the case of the underexpressed genes, a subset of 95 genes are shared by the 3 tumor subtypes (Figure 3B). Additionally, in the case of PTC underexpressed genes, only 72 of the 457 are not shared while almost 400 are shared with FTC (52), ATC (238) or both (95).

### Gene ontology analysis suggests different enriched processes for each carcinoma subtype

For the DEGs on each tumor subtype, GO enrichment analysis was performed, showing significant differences between groups (FTC, PTC and ATC). In Table 2 it is shown how the most enriched categories for each subtype are different:

- In the case of FTC, looking at the overexpressed genes and their associated GO categories, we can see that there are more categories related to cell structure and scaffolding. For PTC, development and extracellular processes are the most enriched categories; whereas for ATC, the most enriched GO categories correspond to cell division.

**Table 2:** **Gene ontology analysis of the differentially expressed genes for FTC, PTC and ATC**

**Subtype (Exp)**	**GO-ID**	**p-value**	**corr p-value**	**x**	**n**	**X**	**Description**
FTC *⇑*	9987	4.8469E-9	1.2888E-5	185	9365	264	cellular process
	44459	2.6863E-8	2.9147E-5	61	1998	264	plasma membrane part
	786	3.2885E-8	2.9147E-5	10	64	264	nucleosome
	32502	2.4620E-7	1.6366E-4	82	3235	264	developmental process
	6334	3.6737E-7	1.9537E-4	10	82	264	nucleosome assembly
	31497	5.7722E-7	2.5580E-4	10	86	264	chromatin assembly
	7155	8.6760E-7	2.6280E-4	29	711	264	cell adhesion
	22610	8.9259E-7	2.6280E-4	29	712	264	biological adhesion
	65004	9.8169E-7	2.6280E-4	10	91	264	protein-DNA complex
							assembly
	30054	9.9358E-7	2.6280E-4	24	521	264	cell junction
PTC *⇑*	5576	8.4568E-20	3.4089E-16	124	2025	471	extracellular region
	32502	2.2428E-18	4.5203E-15	164	3231	471	developmental process
	44421	7.1367E-18	9.5893E-15	77	985	471	extracellular region part
	48731	1.0396E-17	1.0476E-14	134	2420	471	system development
	48856	1.4340E-17	1.1561E-14	142	2653	471	anatomical structure
							development
	7275	1.2464E-15	8.3739E-13	148	2969	471	multicellular organismal
							development
	9611	3.1138E-15	1.7931E-12	51	541	471	response to wounding
	31012	6.5039E-14	3.2771E-11	38	340	471	extracellular matrix
	48513	1.1744E-13	5.2599E-11	101	1790	471	organ development
	48518	1.4424E-13	5.8143E-11	116	2207	471	positive regulation of
							biological process
ATC *⇑*	22403	2.6565E-31	1.6569E-27	105	436	1205	cell cycle phase
	7049	5.2100E-30	1.6247E-26	147	794	1205	cell cycle
	279	1.0473E-29	2.1774E-26	91	352	1205	M phase
	22402	1.9707E-29	3.0727E-26	121	583	1205	cell cycle process
	278	2.8071E-28	3.4578E-25	93	381	1205	mitotic cell cycle
	280	3.8808E-28	3.4578E-25	71	233	1205	nuclear division
	7067	3.8808E-28	3.4578E-25	71	233	1205	mitosis
	87	4.7977E-28	3.7404E-25	72	240	1205	M phase of mitotic cell cycle
	6950	5.0373E-27	3.2463E-24	240	1773	1205	response to stress
	48285	5.2048E-27	3.2463E-24	71	242	1205	organelle fission
FTC *⇓*	44421	3.9892E-20	1.2027E-16	59	984	270	extracellular region part
	5576	1.5267E-18	2.3015E-15	84	2024	270	extracellular region
	5615	3.5497E-16	3.5674E-13	46	746	270	extracellular space
	9653	3.2978E-15	2.4857E-12	58	1216	270	anatomical structure morphogenesis
	48856	2.0741E-14	1.2507E-11	90	2654	270	anatomical structure development
	32502	3.6213E-13	1.8197E-10	99	3232	270	developmental process
	48731	1.5184E-12	6.5398E-10	81	2420	270	system development
	7275	2.0597E-12	7.7626E-10	92	2969	270	multicellular organismal development
	31012	1.4050E-11	4.7067E-9	26	340	270	extracellular matrix
	48513	4.7085E-11	1.4196E-8	64	1790	270	organ development
PTC *⇓*	44421	7.8517E-13	2.8376E-9	61	985	404	extracellular region part
	5615	8.2894E-10	1.0196E-6	46	747	404	extracellular space
	48856	8.4640E-10	1.0196E-6	107	2655	404	anatomical structure development
	48731	2.1381E-9	1.9317E-6	99	2421	404	system development
	5576	2.7064E-9	1.9562E-6	87	2026	404	extracellular region
	9653	4.1464E-9	2.4975E-6	61	1217	404	anatomical structure morphogenesis
	48513	2.6665E-8	1.3767E-5	77	1791	404	organ development
	7275	5.2835E-8	2.3868E-5	110	2970	404	multicellular organismal development
	32502	5.9933E-8	2.4067E-5	117	3233	404	developmental process
	31012	7.5072E-8	2.7131E-5	26	340	404	extracellular matrix
ATC *⇓*	5737	1.2083E-12	7.0080E-9	837	7628	1632	cytoplasm
	5622	1.4044E-9	4.0728E-6	1147	11273	1632	intracellular
	43296	4.8770E-9	9.4289E-6	27	85	1632	apical junction complex
	44444	8.0946E-9	1.1737E-5	574	5140	1632	cytoplasmic part
	16327	1.1319E-8	1.3130E-5	27	88	1632	apicolateral plasma membrane
	70160	2.1881E-8	1.7096E-5	24	74	1632	occluding junction
	5923	2.1881E-8	1.7096E-5	24	74	1632	tight junction
	44424	2.3581E-8	1.7096E-5	1107	10919	1632	intracellular part
	55114	5.1010E-7	3.2873E-4	98	647	1632	oxidation reduction
	5911	6.8676E-7	3.9832E-4	40	192	1632	cell-cell junction

It is shown a set of the top ten categories for each case, overexpressed (*⇑*) and underexpressed (*⇓*).

It is shown the GO-ID, the p-value, adj. p-value, number of genes present in the set and the total of number of genes involved in that GO categorie. Finally, the name of the GO is also shown.
- The underexpressed genes for ATC are related to cell junctions and axes determination. It can be argued that in ATC, the correct function of cell junctions and axis determination are compromised. In the case of FTC and PTC subtypes, extracellular functions, development and cell structure are dampened. This could be so, due to the fact that underexpressed genes are highly shared in both subtypes.

### Pathways Analysis for each carcinoma subtype

Different features for all the networks associated to cell death and survival and cell to cell signaling processes involving DEGs and mined from each pathway were examined using IPA: preferential compartmentalization, highly connected molecules (hubs) and differentially expressed molecules (Table 3).

**Table 3:** **IPA analysis of signaling pathways involved in cell-death and cell-to-cell signaling events**

**Category**	**Subtype**	**Compartment**	**Hubs**	**DEGs**
**Cell Death**	*ATC*	M, C, N	*MMP1+, MMP3+, JNK-, FOXO1-, CASP3+, CASP7+*	*MMP1+, NUSAP1+, DEPDC1+, SPINT2-*
	*FTC*	M	BCR*, NFKB*, ADRB2-	*FOSB-, CCL2-, LPL+*
		C	IL12*, IFA*, CD3*, CD44+, BAX+, HSP70*, JUN-	*JUN-, CD44+*
		N	CTNNB1*, KCNIP4-, PGR*	*GDF15+, KLF4-, SLIT2-*
		N	AKT*, E2F1*, TP73*, E2F*	*SFRP2-, GATA5+*
	*PTC*	M, C	CG*, FSH*,LH*	*PPARGC1A-, SLC4A4-, HMGA2+*
		M, C	PI3K family*, AKT*,BCR*, IGs*	*PROM1-, IGSF1+*
		C	TGM2+, Caspases*	*TIMP1+, SLC25A15, FHL1-*
		N	CD44+, AKT1*, GLI1*, POU5F1*	*PAPSS2-*
**Cell-Cell Signaling**	*ATC*	M	CG*, Secretase gamma*, PLAU+	*PLAU+, PLAUR+, SPP1+, LRP1B-,PPAP2B-, SLC4A4-*
		E	*IL1B+, CCL20+, IL1RN+*	*TSHR-, PDE8B-, FCGR2A+, TNFA1B6+, TG-*
		M	IFNA*, IFNB*, IL12*, MYD88+	*CXCL5+, CD86+, CLEC7A+*
		M	*IL8+, CD14+, TLR2+*	*IL8+, CCL21-, TLR2+*
	*FTC*	E, M, C	IL8*, IL13*, COL18A1*, HB*, CBL*	*ZMAT3+, GJB6-, EPHB1-,EPHA3-*
		M	Estrogen receptor*, ITGB1*, CD46*, SPDEF*,FOXA1*	*COL13A1+, CDH16-*
		M	*{ }*	*XPR1+*
	*PTC*	M, C	NFKB*, GH*	*CITED2-,GHR-, CDH3+*
		M, C	MAPK1*, F2*, HSF1*	*FN1+, SHANK2-*
		E	IFNG*, IL1A*, p73*	*SERPINA1+, CLMN-*

It is shown the compartment in which the found molecules are more abundant (M for plasma membrane; C for cytoplasm; N for nucleus and E for Extracellular Matrix), The most connected molecules (hubs) and the most differentially expressed genes. The sign (+,- and *) represent overexpression, underexpression and no-change with respect to control.

IPA®; generates by default the top 25 networks according to the number of molecules resulted from the analysis. These networks are divided by general functions. Networks in which the cell-death and survival as well as cell-cell-signaling mechanisms are enriched were chosen. This is due to the fact these mechanisms are highly related to tumor progression and invasiveness.

#### Cell death and survival

*Follicular Thyroid Carcinoma.* The most important features shared among the FTC networks in cell death and survival processes are as follows: one of the networks is mainly regulated by BCR, the others involve either CTNNB1, the AKT-TP73 pathway or CD44-BAX-JUN. Molecules are mainly located in the nucleus. Top DEGs for each network were FOSB-, CCL2-, LPL+, JUN-, CD44+, GDF15+, KLF4-, SLIT2-, SFRP2-, GATA5+ (Additional file 2).

*Papillary Thyroid Carcinoma.* In the case of PTC, the most important features shared among networks in cell death and survival are as follows: One of the networks is mainly regulated by the female hormones (Cg, FSH and LH), the others mainly involve caspases, AKT pathway or CD44. Molecules are fundamentally located in the plasma membrane and cytoplasm. Top DEGs for each network were PPARGC1A-, SLC4A4-, HMGA2+, PROM1-, IGSF1+, TIMP1+, SLC25A15, FHL1-, PAPSS2- (Additional file 2).

**Figure 4:**
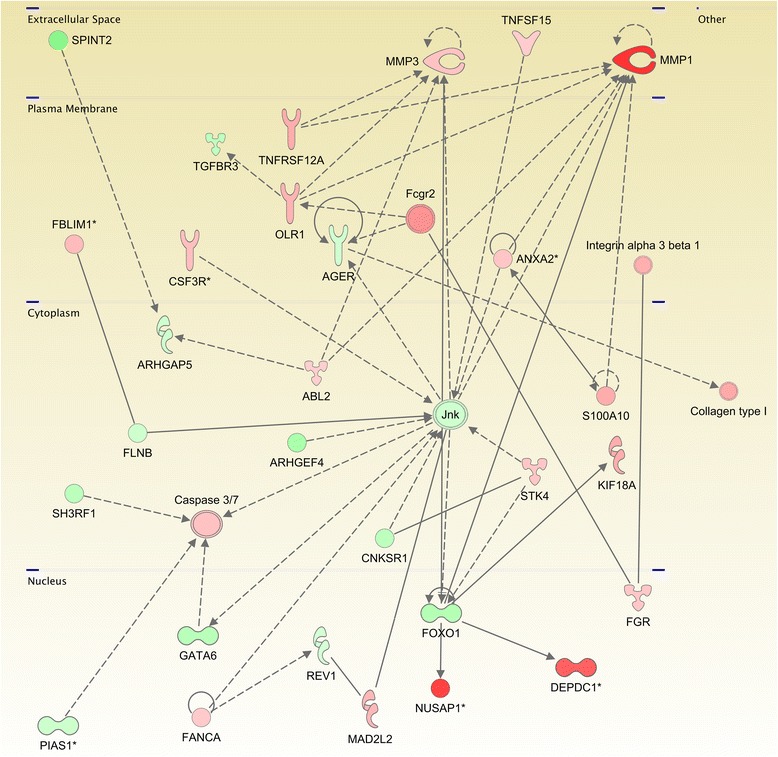
**Pathway for a network related to cell-death and survival process in the anaplastic thyroid carcinoma.** This graph shows the interaction network of the differentialy expressed genes of ATC which have a role in death and survival events. Red molecules are overexpressed and green molecules shows underexpression. Color intensity represents the difference between the ATC samples compared with the normal ones. Notice the high degree of conection of MMP1 and Jnk molecules. It is also worth to mention that most molecules in the plasma membrane are overexpressed meanwhile those in nucleus and cytoplasm have low expression values.

*Anaplastic Thyroid Carcinoma.* There is only one significant network for cell death and surival in ATC: As it can be observed in Figure 4, it is mainly regulated for metalloproteinases, caspases, tumor necrosis factor receptor and tyrosine kinase activities. Figure 4 represents the molecules involved in a subpathway related to cell death and survival signaling pathway (as annotated in IPA/IKB), and are also present in the list of differentially expressed genes in ATC samples for both, underexpressed as well as overexpressed. All molecules are depicted according to their cellular localization. The links binding molecules represent experimentally-verified relationships in the IKB (See “Methods/Pathway Analysis” subsection). The figure illustrates how MMP’s are overexpressed mainly because their regulators are also overexpressed and how caspases 3 and 7 are overexpressed because their negative regulators are underexpressed.

Figure 4 also shows how MMP1, NUSAP1 and DEPDC1 are the most overexpressed genes. Concerning NUSAP1 and DEPDC1, the only DEG that regulates them is FOXO1, a very ubiquitous transcription factor. In the case of matrix metalloproteinase 1, there are several regulators, most of them (with the exception of FOXO1 and Jnk) also overexpressed. The fact that MMP1 and MMP3 are highly overexpressed suggest that the mechanisms for migration and invasiveness may be active. On the other hand, NUSAP1 is a nucleolar-spindle-associated protein that plays a role in spindle microtubule organization. High levels of this protein have been observed in cervical cancer [31]. DEPDC1 might play an essential role in the growth of bladder cancer cells [32]. DEPDC1A knockdown delayed the growth of human myeloma cell lines (HMCLs), with a block in G2 phase of the cell cycle, p53 phosphorylation and stabilization, and p21 (Cip1) accumulation [33]. Hence DEPDC1A overexpression in ATC may be related with the growth of cancer cells. Other interesting features are that KIF18, a member of the kinesin superfamily of microtubule-associated molecular motors is overexpressed whereas SPINT2 is a putative tumor suppressor that appears to be underexpressed.

IPA/IKB algorithm for CN was applied as follows: First, the list of molecules in the cell death and survival subpathway were analyzed seeking statistical enrichment in our DEG set. After statistical significance was achieved, the algorithm retrieves a list of experimentally verified interactions from the master network and depicted them in a pathway color-coded according to the average fold-changes in the GEA for the contrast under consideration.

#### Cell-to-cell signaling

*Follicular Thyroid Carcinoma.* In these networks none of the hubs are differentially expressed in the pathways. Plasma membrane is the most important compartment. Top DEGs are: ZMAT3+, GJB6-, EPHB1-,EPHA3-, COL13A1+, CDH16- and XPR1 (Additional file 2).

*Papillary Thyroid Carcinoma.* Again, there are no differentially expressed hubs in PTC. Plasma membrane is once more the most important compartment. The top DEGs are CITED2-,GHR-, CDH3+, FN1+, SHANK2-, SERPINA1+, CLMN- (Additional file 2).

**Figure 5:**
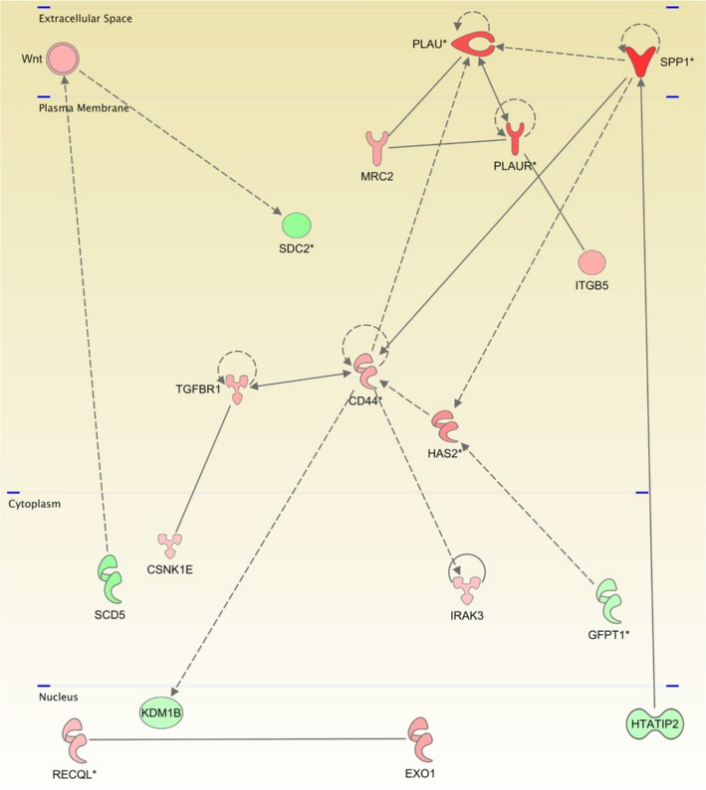
**Pathway for a network related to cell to cell signaling process in the anaplastic thyroid carcinoma.** Please notice the overexpression of PLAU, PLAUR and SPP1.

*Anaplastic Thyroid Carcinoma.* In contrast, all hubs are DEGs and perhaps more outstanding, all of them are overexpressed: PLAU+, IL1B+, CCL20+, IL1RN+, MYD88+, IL8+, CD14+, TLR2+. All molecules signalize in plasma membrane or extracellular matrix. Finally, the top DEGs are PLAU+, PLAUR+, SPP1+, LRP1B-,PPAP2B-, SLC4A4-, TSHR-, PDE8B-, FCGR2A+, TNFA1B6+, TG-, CXCL5+, CD86+, CLEC7A+, IL8+, CCL21-, TLR2+. For ATC most of the signaling molecules are receptors. It seems that endocrine-and-paracrine-related signaling is more prevalent than in FTC or PTC. Interestingly enough, even when, in general, ATC possess a higher number of underexpressed genes (1917 vs. 1303 overexpressed), most molecules in ATC-related signaling pathways are overexpressed, pointing out to exacerbated extracellular signaling in ATC (Figure 5).

### Pathway commons analysis from WEB-gestalt

**Figure 6:**
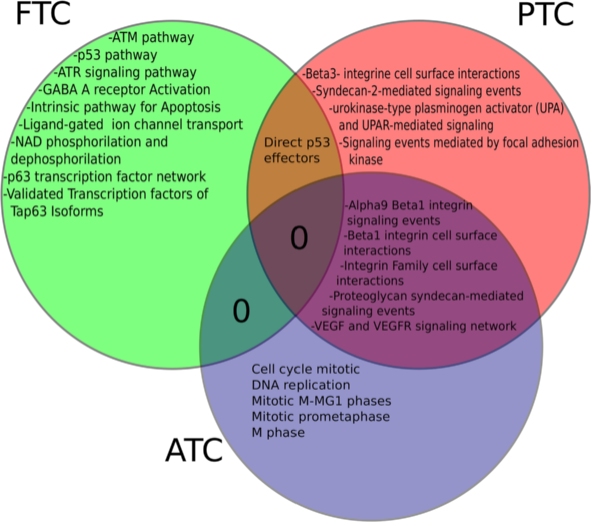
**Venn diagrams for shared pathways from the WebGestalt for common pathways analysis.** It is remarkable that the non-shared pathways for FTC (the less malignant) are related to DNA repairing and Regulation of cell cycle; for PTC the paracrine events and for ATC the mitosis pathways. The shared pathways also reflects the inter-type events. Again, FTC-PTC share Direct p53 effectors, meanwhile PTC-ATC share ce–to-cell signaling pathways as well as grow factor-related events.

An important result is the set of shared pathways among the different subtypes of carcinomas (Figure 6). While FTC and PTC only share the P53 effectors pathway, PTC and ATC share *α*9*β*1 integrin signaling event; *β*1 integrin cell surface interactions, integrin family surface interactions, proteoglycan syndecan-mediated signaling events and finally VEGF & VEGFR signaling network. Enrichment analysis of this gene list shows three main pathways: ATR signaling pathway (adj_p-value= 0.0413), integrins in angiogenesis (adj_p-value= 0.0413) and ATM pathway (adj_p-value= 0.0452). For a detailed account on the enriched categories exclusive to each tumor subtype see the Discussion Section.

### Enriched pathway commons from IPA

**Figure 7:**
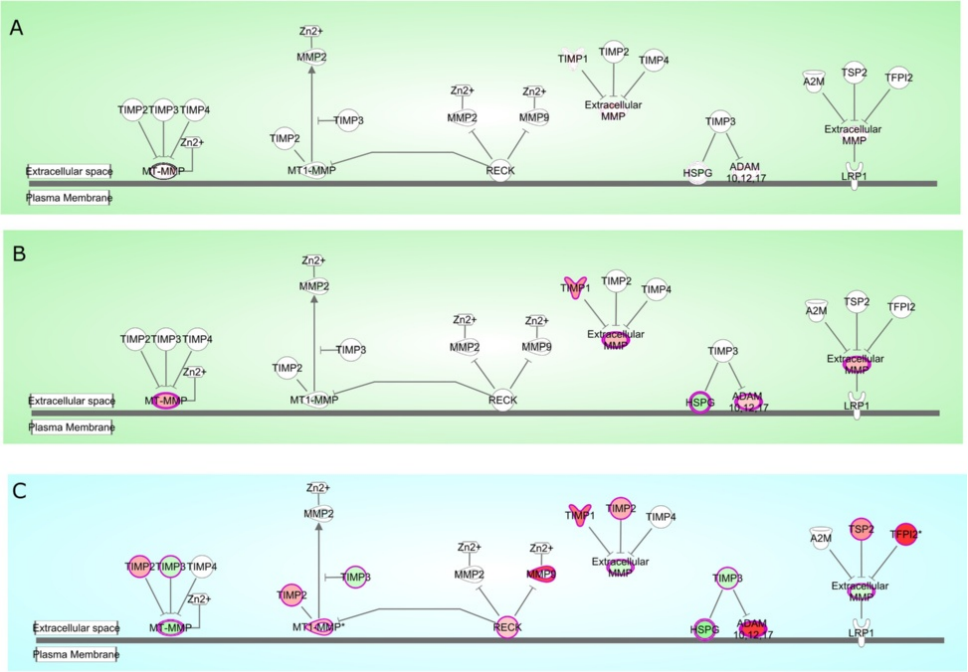
**Inhibition of matrix metalloproteinases pathway for each case: A) FTC, B) PTC and C) ATC.** It is shown the contrast in the differentially expressed genes for each case. FTC does not have DEGs, PTC has only a few but ATC has very high differentially expressed genes, specially, ADAM, TIMP and MMPs, suggesting a very important role of this pathway in the progression of ATC.

IPA was used to investigate the pathways shared by the DEGs from FTC, PTC and ATC. It is important to mention that the most enriched pathway in ATC was the one related to Inhibition of Matrix Metalloproteinases (IMM). PTC, however, is poorly enriched and FTC is completely absent (Figure 7) This might be indicative of invasiveness enhancement, migration, colonization and metastasis in ATC with respect to PTC or FTC.

## Validation

It is known that the cornerstone of science (and in particular of computational biology) is validation of the results and alternative hypothesis testing. This is specially true in the case of high throughput gene expression analyses like those performed in this work. In order to comply with such an important requirement we implemented the same analysis pipeline we follow in our original experimental design in two independent thyroid cancer gene expression datasets.

In this section we will present results of the validation procedures related to our main findings in this work. The complete results for gene expression analysis for the validation sets (and also for the original dataset) as well as other related calculations are included as supplementary material.

In order to validate the results obtained by the differential expressions analysis in our dataset, we perform differential analysis on two independent datasets. In the case of the top differentially expressed genes, we found that for those genes the expression levels resulted in agreement with our original analysis in 27 out of 30 comparisons. Results are shown in Table 4.

**Table 4:** **Validation of top differentially expressed genes in PTC, FTC and ATC with two independent datasets**

**Subtype**	**Gene name**	**Set 1-Validated**	**Set 2-Validated**
**FTC** ***⇑***	ZCCHC12	yes	yes
	PRR15	yes	yes
	CA12	yes	yes
	TENM1	yes	**no**
	ARHGAP36	**no**	**no**
**FTC** ***⇓***	ADH1B	yes	yes
	DCN	yes	yes
	CCL21	yes	yes
	CHRDL1	yes	yes
	DPT	yes	yes
**PTC** ***⇑***	GABRB2	yes	yes
	HMGA2	yes	yes
	PRR15	yes	yes
	CHI3L1	yes	yes
	ZCCHC12	yes	yes
**PTC** ***⇓***	PKHD1L1	yes	yes
	TFF3	yes	yes
	TPO	yes	yes
	DIO1	yes	yes
	ADH1B1	yes	yes
**ATC** ***⇑***	POSTN	yes	yes
	MMP1	yes	yes
	SPP1	yes	yes
	TFPI2	yes	yes
	VCAN	yes	yes
**ATC** ***⇓***	DIO1	yes	yes
	TSHR	yes	yes
	TG	yes	yes
	SLC26A7	yes	yes
	TPO	yes	yes

For this analyisis the 5 top overexpressed and underexpressed genes of our datasets was tested against two independent genome-wide gene expression databases (available in Additional file 4). The first column shows over/under-expression of each gene within our set; second column shows the gene names. Gene validation status (cols. 3 and 4) shows that 27 out of 30 relationships were consistent with our original analysis.

**Figure 8:**
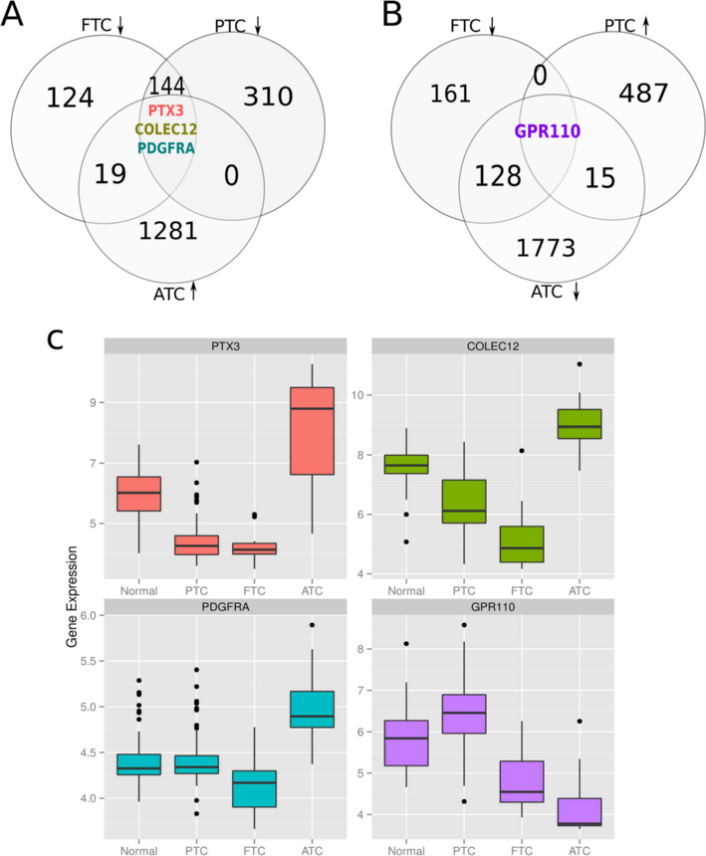
**Possible biomarkers to differentiate among thyroid carcinomas.** **A)** Venn diagrams which contain the overexpressed genes of ATC together with those which are underexpressed in FTC and PTC. **B)** follows the same logic but the overexpressed genes belong to PTC meanwhile the underexpressed are for ATC-FTC. **C)** Boxplot of the expression levels of those genes for each case with respect to control.

**Figure 9:**
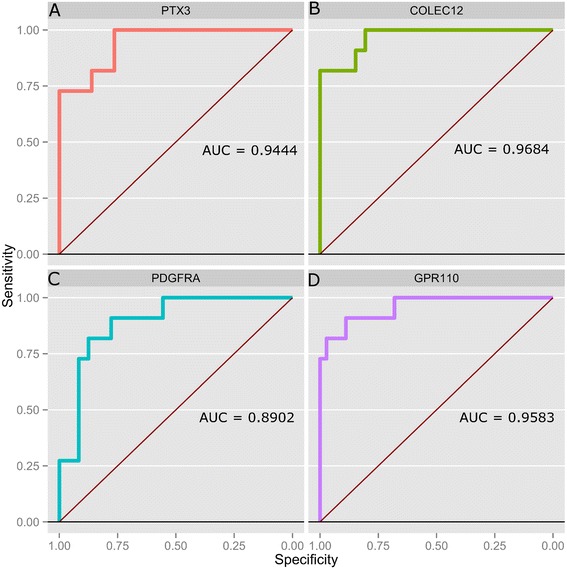
**ROC curves for the possible biomarkers to differentiate among thyroid carcinomas.** For each molecule it is showed the ROC curve as well as the Area Under the Curve (AUC) value. The color code is the same than in Figure 8. **A)** curve for PTX3; **B)** COLEC12; **C)** PDGFRA. Those 3 genes were overexpressed in ATC meanwhile underexpressed in PTC. **D)** shows GPR110 ROC curve, which resulted overexpressed in PTC and underexpressed for ATC. Results are in full agreement with those in Figure 8.

Regarding the presence of genes whose differential expression profiles may provide us with a means of molecular differentiation between subtypes (i.e. candidate biomarker molecules), we found four of them PTX3, COLEC12, and PDGFRA (whose overexpression is likely indicative of an ATC phenotype) and GPR110 (whose overexpression may indicate PTC). We validated those gene expression profiles by a comparison of their actual probability distribution across samples in the validation sets. Boxplot-outlier analysis as well as Receiver-Operator Characteristic and Area-under-the-curve calculations (ROC-curves and AUC respectively) for the validation datasets were performed in an identical manner to the analysis in Figures 8 and 9. Results were consistent (Table 5), detailed plots are included as Additional files 3 and 4.

**Table 5:** **Validation profile for the candidate biomarker genes PTX3, COLEC12, PDGFRA and GPR110 in two independent experimental datasets**

**Subtype**	**Dataset 1 (original)**	**Dataset 2 (validation 1)**	**Dataset 3 (validation 2)**
PTX3 expression profile	Original profile	Consistent	Consistent
COLEC12 expression profile	Original profile	Consistent	Consistent
PDGFRA expression profile	Original profile	Consistent	Consistent
GPR110 expression profile	Original profile	Consistent	Consistent

Validation of the Gene expression profile for PTX3, COLEC12, PDGFRA and GPR110 in two independent datasets. First column shows gene names, second column refers to the original profile as in Figure 8, third and fourth columns refer to the validation status respect to the original profile.

Regarding the phenomenon of dysregulation of the Inhibition of Matrix Metalloproteinases Pathway in ATC we perform IPA/IKB causal network calculations (see the Pathway Analysis subsection in Methods) for the two independent validation datasets to check whether this was the case. The results for the three tumor subtypes are presented in Table 6. As one can see there, IPA/IKB pathway analysis for FTC and PTC show no statistically significant dysregulation of this pathway (as expected). In the case of ATC, this pathway resulted dysregulated, reaching significance level in two out of three experimental datasets.

**Table 6:** **Validation of the status of the inhibition of matrix metalloproteinases pathway in PTC and ATC with other sample subset**

**Subtype**	**Dataset 1**	**Dataset 2**	**Dataset 3**
	**(original)**	**(validation 1)**	**(validation 2)**
FTC	Not significant	Not significant	Not significant
PTC	Not significant	Not significant	Not significant
ATC	**Active**	Not significant	**Active**

For this IPA/IKB analyisis, the Inhibition of Matrix Metalloproteinases (IMMPP) pathway was tested against two independent genome-wide gene expression databases. The IMMPP resulted with no statistical significance (at the *p* −*value* = 0.01 level) for both FTC and PTC in all three cases (original dataset and two independent validation datasets) as expected. However, despite the statistical significance of the whole pathway, some molecules related to IMMPP in PTC resulted differentially expressed (depicted in Figure 7B). In the case of ATC, the IMMPP resulted dysregulated. However only two out of three experimental datasets (the original and one of the validation sets) reached statistical significance (*p* −*value* ≃ 10^−8^ and ≃ 10^−5^, respectively).

Datasets chosen for validation are publicly available and reported in Additional file 4. It is worth mentioning that although it would be highly desirable to have higher sample counts for the inference and validation of results, at the time there is no public availability of larger experimental datasets. Future lines of inquiry would be by necessity associated with the development larger whole genome gene expression experimental datasets for thyroid neoplasms.

## Discussion

During this research, a model of thyroid carcinoma progression based on genome-wide expression datasets has been built by using three different approaches and two validation sets. According to the differentially expressed genes obtained in our samples, we suggest differences between the three cancer subtypes at the molecular level have an important impact on signaling. This impact induces observed phenotypic differences. Using WEBGestalt (see “Methods”) for dissecting pathways, the main biological features associated with the DEGs were dissected for each subtype. By using IPA, pathways and their relations were able to be observed, mainly those in which the DEGs participate deepening knowledge on the mechanistic functions of the molecules involved in each subtype. Furthermore, integrating three analysis tools proved to be useful for an insight into the relevancy of some molecules on the progression of thyroid carcinoma; mainly, molecules involved in cell to cell signaling events as well as cell death and survival cell-death processes, namely, the metalloproteinases, the caspases and their regulators. Moreover, the concomitant effects that several pathways have on certain key molecules related to cell-death and migration events were determined.

### Invasiveness and migration events in ATC could be exacerbated by synergistic pathways

In addition, insight was achieved regarding processes related to growth of tumor mass and tissue invasion and the degradative action of extracellular MMP or membrane-type MMP (MT-MMP) subfamily. Proteins encoded by MMP and MT-MMP genes can alter cell-cell and cell-ECM junctions; both mediate the release and activation of autocrine or paracrine signaling molecules such as angiogenic activators as well as activate or deactivate cell surface receptors, promoting invasiveness and metastasis [34]. On the other hand, matrix metalloproteinase-1 may be responsible for the aggressiveness or bone metastasis of poorly differentiated thyroid carcinoma [35].

Proteolytic activities of MMP and MT-MMP are tightly regulated by the family of tissue inhibitors of metalloproteinases (TIMP) in a reversible fashion [36,37]. Unlike TIMP1, TIMP2, and TIMP4, which are secreted in soluble form, TIMP3 is present only in association with ECM. TIMP3 has been shown to promote apoptosis whereas TIMP1 is active in blocking apoptosis. Overexpression of TIMP2 protects cancer cells from apoptosis [38,39].

Some MMP have anti-angiogenic action, such as metalloproteinase disintegrin activity thrombospondin motif (ADAMTS). This subgroup of MMP has thrombospondin-like domains, and it has been observed that some of its members are able to block the activation of VEGF-induced angiogenesis [40]. Members of the ADAM family are cell surface proteins with a unique structure possessing both potential adhesion and protease domains. ADAMTS encodes a protein that cleaves many other proteins including TNF- *α* and E-cadherin.

In addition, other proteins that act as inhibitors of MMPs have been described, noting that some contain domains homologous to domains of the TIMPs. For example, the reversal inducing protein cysteine-rich motif Kazal (RECK) is an inhibitor that has a regulatory role in the integrity of the extracellular matrix in angiogenesis [41] that is overexpressed in ATC.

On the other hand, taking into account the molecules involved in cell to cell signaling events, the most DEG was osteopontin (SPP1), which has been found disregulated in several subtypes of cancer [42] which in turn is linked to PLAU and PLAUR, also highly overexpressed. Nearly all normal samples were underexpressed for SPP1. Some thyroid adenomas have been found weakly SPP1 positive, whereas many carcinomas were strongly positive [43].

As it has been noted on previous work [44]: CST6, CXCL14, DHRS3, and SPP1 are regulated by BRAF signaling and may play a role in papillary thyroid carcinoma pathogenesis. We suggest that SPP1 might be used as a candidate diagnostic and prognostic marker for these tumors. Furthermore, given the role of the SPP1-CD44v6 axis in PTC cells, it has been suggested that CD44 and/or SPP1 may be molecular targets for therapeutic intervention in aggressive PTCs [45]. PLAU, a serine protease involved in degradation of the extracellular matrix and possibly tumor cell migration and proliferation [46,47] was also overexpressed in our ATC data.

Despite the fact that some previous results point out to SPP1-related cell signaling in PTC, we found SPP1 as one of the most DEGs in ATC. Taking into account that the most differentially expressed gene involved in cell death and survival was MMP1 –a protein also related to migration and adhesion– (Figure 4) and the most DEG in cell to cell signaling was SPP1 (Figure 5), we were also able to establish that invasiveness, migration and proliferation via PLAU and MMP could occur autonomously and they may exert an increased synergistic proliferative phenotype; thus, increasing aggressiveness.

From our results, LRP is underexpressed in ATC. Low expression of LRP at the cell surface was associated to elevated extracellular MMP2 and urokinase plasminogen activator (uPA) activities as well as to high invasiveness properties [2]. Thyroid carcinoma aggressiveness was widely increased by exogenous uPA. Anti-uPA antibody treatments abolished both basal and receptor-associated protein-induced thyroid cell invasion [48]. Overall, our results are consistent with the fact that, in the most aggressive carcinoma (ATC), LRP gene is underexpressed, which could favor thyroid carcinoma cell invasion.

### Inferred progression FTC-PTC-ATC

The common-pathway-analysis obtained by the WEBGestalt tool showed some shared networks in the three subtypes of carcinoma. This sharing between the different subtypes results revealing, because it is possible to try to establish a timeline of progression related with the main activities of those pathways, since the most enriched pathways for each cancer subtype lies on different categories which in turn are related to different processes in the cell. These processes are associated to distinct moments in the cell’s lifespan.

Starting with FTC, the less aggressive carcinoma, the principal functionalities are related to DNA damage repair and cell cycle regulation; i.e. the repairing machinery is available and works to face damage of cancer cells. This cancer subtype shares *Direct p53 effectors* pathway with PTC subtype. For PTC the most enriched pathways found was mainly related to cell to cell signaling events and adhesion to extracellular matrix; those pathways seem to be involved in events of paracrine signaling, suggesting an incipient invasion of cancer cells, but without other hallmarks of cancer, like evasion of apoptosis or immortalization. Finally, PTC shares with ATC some cell to cell signaling events as well as mitosis processes (*VEGF and VEGFR signaling network* [49]). The rest of enriched categories in ATC are focused on cell cycle progression, indicating metastasis and invasiveness, thus suggesting FTC → PTC → ATC progression.

Further support for this assertion lies in the fact that in most cases ATC arise in association with a differentiated carcinoma –mainly PTC– as well as some poorly differentiated lesions [2,5,14]. Hurtle cell tumors and other follicular carcinomas are also known to be able to undergo anaplastic transformation [2,14].

In PTC, aberrant methylation of tumor suppressor genes such as TIMP3 and DAPK, has been associated with tumor aggressiveness [11]. TIMP1 and TIMP3 has been reported to be underexpressed in PTC and FTC, respectively [11]. During our analysis, DAPK1 and DAPK2 are underexpressed in FTC and ATC, respectively, but not in PTC. However, contrary to data obtained by Hu et al. [11], TIMP1 is overexpressed in our PTC samples (as is also in ATC).

Gene expression analysis has previously demonstrated that cyclin D1 (CCND1) is overexpressed in PTC [12]. In our data CCND1 is overexpressed in PTC and FTC as it was previously discussed. CCND2 is underexpressed in ATC and there are no overexpressed CCNDs in anaplastic thyroid carcinoma, suggesting an abscence of progression of the cell-cycle via cyclins in ATC.

### Possible crosstalk in cell-death-and-survival events could be involved in the transition from PTC to ATC

**Figure 10:**
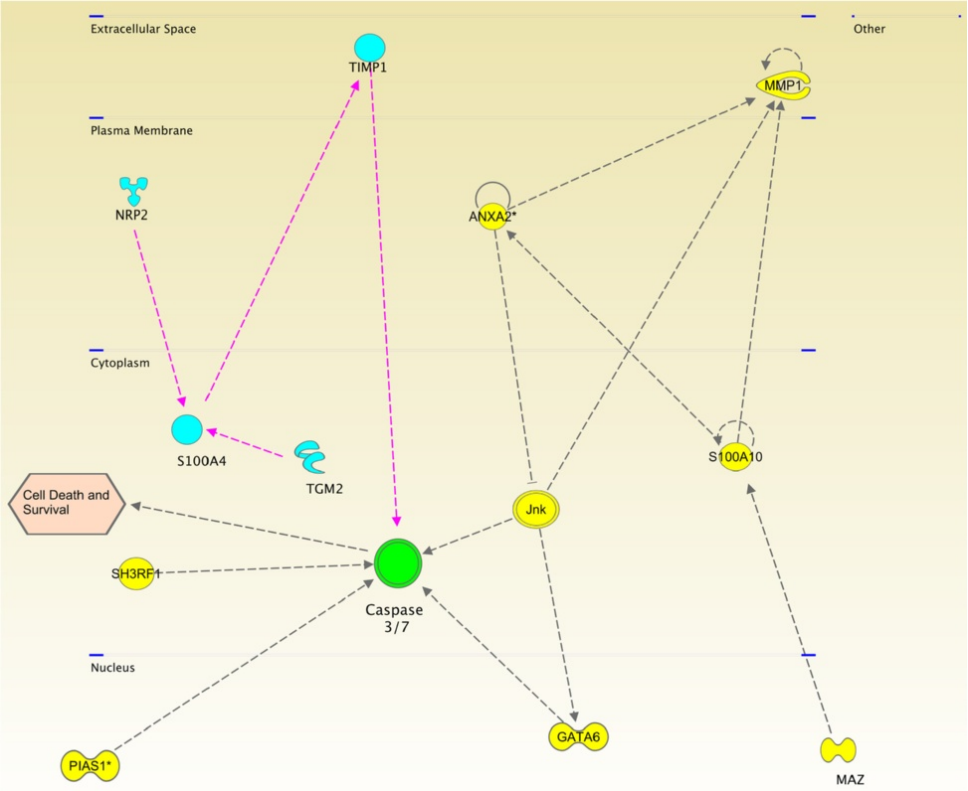
**Relevant crosstalk of altered signaling pathways involved in cell death for PTC and ATC.** Molecules enriched in PTC are depicted in turquoise meanwhile those enriched in ATC are depicted in yellow. Notice that both pathways start in both intra and extracellular space and also both culminate with the inhibition of caspases.

Enriched pathways of cell death and survival events contain molecules relevant for each subtype of carcinoma. However, different tumor subtypes can share some elements, indicating that shared molecules participate in both subtypes, possibly in a different fashion. In our case, caspases 3 and 7 were overexpressed as well as both PTC and ATC, being also regulated by distinct molecules and showing a different expression levels. For the case of ATC, caspases 3,7 were shown being regulated by Jnk, GATA6, SH3RF1 and PIAS1: all of them underexpressed. On the other hand, PTC presents caspases 3,7 at normal expression levels and being regulated by TIMP1. TIMP1 and its regulators are overexpressed, making thus a difference in the mechanisms of action of caspases. However, TIMP1 is also overexpressed in ATC, suggesting that the disregulation of the caspases activity could be determined by changes of its modulators. Furthermore, this crosstalk between PTC and ATC pathways may difficult the action of therapeutics against caspases, since they can be multiple-modulated (Figure 10).

Our results are supported by several other experiments as well as recent literature. For example, LGALS3 (galectin-3) is overexpressed in ATC and PTC, as it is mentioned by Wiseman et al. [49], as well as p16 (CDKN2A) and HMGA2. Changes in gene expression profiles for LGALS3 provide insights into stages of tumor progression. For example, a study of 100 benign and 105 malignant thyroid tumors using a panel of 57 molecular markers found an association of tumor pathology with the expression of a subset of markers including cytokeratin 19 (CK19), LGALS3, HBME-1, VEGF, AR, p16, and AURKA [49].

As it was pointed above, expression of HMGA2 correlates with malignant penotype of thyroid tumors and can be used as a tool to differentiate malignant from benign lesions [50,51]. HMGA2 was found overexpressed in the three cases, reinforcing its role to differentiate.

### Pathway dissection for cell-to-cell signaling

Since a significant difference among the differentially expressed molecules involved in the IMM pathway was found for each subtype of carcinoma, we decided to dissect thoroughly the cell-to-cell singling pathways. Our analysis showed the following:

- **FTC:** Metalloproteinases and TIMP3 are underexpressed, indicating an absence of migration or invasiveness. On the other hand, the Bax-pathway is also inhibited, which may be indicative of an absence of apoptotic processes.
- **PTC:** MMP1 is overexpressed, but its inhibitor TIMP1 is also overexpressed, suggesting a competition for the progression of invasiveness. Caspases are overexpressed too, thus leading to conflicting signals of cell death and progression.
- **ATC:** One of the most differentially expressed molecules in the whole analysis was the metalloproteinase MMP3 suggesting migration and invasion processes. Furthermore, caspases 3 and 7 are also overexpressed, possibly due to the downregulation of Jnk via ANXA2 inhibition.

### DEGs downregulate some pathways meanwhile increase others

After finding sets of shared underexpressed genes, we found only 11 genes involved in 10 pathways. The genes in question are CHRDL1, EGR2, FGFR2, FOSB, IPCEF1, JUN, KCNIP4, NCAM1, SCN3B, TBC1D4 and TGFRB3. The associated pathways are the following:

1. EGF-receptor ERB1 signaling pathways
2. IL-3-mediated signaling events
3. Glypican pathway
4. LKB1 signaling events
5. Endothelins pathway
6. Class I PI3K signaling events mediated by Akt
7. Arf6 trafficking events
8. Arf6 downstream pathway
9. PDGFRB signaling pathways
10. Class I PI3K signaling events

Those pathways seem to be dampenened in some manner in all three cancer subtypes.

Like, the set of 35 overexpressed shared genes present only 1 common pathway: Integrin family cell surface interactions, with 5 genes: GDF5, EDIL3, ITGA1, TNFRSF10B and EPS8. To our knowledge, none of these genes have been found associated to thyroid carcinomas. We noticed as well that PDGFRA could serve as a biomarker for ATC, since it is underexpressed in FTC and PTC. It is the same case for COLEC12 and PTX3.

### Possible candidate biomarkers to differentiate among thyroid carcinomas

ATC is quite different at the gene expression level than the other two tumors. ATC is the cancer subtype with more underexpressed genes than overexpressed ones. Comparison of genes overexpressed in ATC while underexpressed in FTC and PTC are PTX3, COLEC12 and PDGFRA (Figure 8A). DNA microarray analysis has shown platelet-dervived growth factor receptor (PDGFR) overexpression in ATC relative to well-differentiated thyroid cancer [52]. Interestingly enough PDGFRA promotes lymphatic metastases in papillary thyroid cancer [53]. Also, inhibitors of PDGFR can supress anaplastic thyroid carcinomas [54]. Both Akt and the PDGF/PDGFR system play crucial roles in cell proliferation, differentiation, migration, invasion and tumorigenesis, and development and metastasis of ATC. These findings imply that autocrine activation of PDGF- *α* receptor plays a crucial role in the carcinogenesis of thyroid cells [55]. GPR110 in turn is overexpressed in PTC while underexpressed in ATC and FTC (Figure 8B). GPR110 has been hypothesized to be oncogenic and has been observed overexpressed in lung and prostate cancer [56]. PTX3 is an inflammatory factor involved in fertility [57] and also, its overexpression has been associated to lung cancer [58] (Figures 8C and 9). Validation for this results are showed in the supplementary material.

### Summary of main findings

By means of a genome-wide analysis, differentially expressed genes were found for three different subtypes of thyroid carcinoma: Follicular (FTC), Papillar (PTC) and Anaplastic (ATC). By analyzing their differences we have pointed out a possible progression from FTC to PTC and after that to ATC. We also have shown that those three subtypes share both overexpressed and underexpressed sets of genes determining that most enriched pathways for each subtype of carcinoma are different and these differences, could be related to the aggressiveness and invasiveness. Finally, an interesting result obtained by the pathway analysis is that metalloproteinases disregulation could be involved in the progression from PTC to ATC in an irreversible fashion; the underexpression of TIMP3 gene in ATC could be responsible for the matrix metalloproteinases inhibition pathway being unregulated, thus facilitating invasiveness and the concomitant metastasis. Moreover, overexpression of PLAU, PLAUR and SPP1 could exert an increased cell-to-cell interactions making thus an enhanced metastatic effect.

Regarding the set of differential genes that we suggest may constitute candidate biomarkers (PTX3, COLEC12 and PDGFRA for ATC and GPR110 for PTC), one thing could be said: since these molecules have been scarcely studied they may well constitute interesting targets for directed research in relation to thyroid carcinoma.

In an attempt for summarize our findings we can state the following:

1. There is evidence to suggestting progression of thyroid carcinoma could happen from FTC to PTC and finally to the undifferentiated ATC.
2. FTC and PTC share some characteristics –genes and pathways– which are related to DNA damage repair or cell cycle arrest. At the same time, PTC and ATC share pathways and genes related to cell to cell signaling.
3. PTX3, COLEC12 and PDGFRA could be used as biomarkers to differentiate ATC from FTC or PTC since these 3 genes are overexpressed in ATC meanwhile underexpressed in the other two subtypes.

## Conclusions

Regulation of biochemical pathways in cancer is an exciting and ever changing field. A number of challenges need to be overcome though. In the case of undifferentiated thyroid carcinoma there is a still a need for comprehensive genome-wide studies in larger sample databases. The required amount of information typical of current Systems Biology studies demands that more and more.

Pathway crosstalk is one of the biggest difficulties to perform correct therapies. This work is an attempt to dissect important pathways with the aim to find crosstalk points in order to develop more specific drugs. Even when hundreds of genes resulted differentially expressed in tumor samples, this study focused on those genes in which the most enriched pathways are involved, to distant ourselves from a gene-centric approach in favor of a pathway-centered mechanistic analysis. More work on the functionality of DEGs and their related pathways in such complex phenotypes is needed.

## Additional files

Additional file 1
**Differentially expressed genes (DEGs).** Each file shows the name, gene symbol and affyID, logFoldChange, Average Expression, t-statistic, P value, adjusted P value y B statistic for each gene. Genes with (*L* *F* *C*)>1 and *B*−*s* *t* *a* *t* *i* *s* *t* *i* *c* *s*>5 were chosen to perform the analysis. Sheet1 for ATC. Sheet2 for FTC and sheet3 for PTC.

Additional file 2
**Figures of the pathways of cell-to-cell signaling as well as cell-death processes, obtained by the DEGs using IPA software.** Each figure shows the molecules that were differentially expressed depending on the involved pathway.

Additional file 3
**ROC curves for the possible biomarkers obtained with the validation subsets.** It is showed the same color pattern than Figure 9, as well as the ROC value of each case. It is important to mention that for all cases the results are according to those obtained by Figure 9.

Additional file 4
**Validation files.** A compressed folder that includes two subfolders (named ValidationSet1 and ValidationSet2, respectively). Those folders include the following files:.csv files with the results of the gene expression differential analysis for the three contrasts (ATC vs normal thyroid tissue, FTC vs normal thyroid tissue and PTC vs normal thyroid tissue)..csv files with the normalized expression matrix for the preprocessing of the validation arrays (each array is identified by its GEO accesion key). PDF files of the volcano plots for the differential expression analysis.
