# Supplementary figures and images for: Genome-wide expression analysis suggests a crucial role of dysregulation of matrix metalloproteinases pathway in undifferentiated thyroid carcinoma
Source: BMC Genomics. 2015 Mar 18;16(1):207. doi: 10.1186/s12864-015-1372-0 (PMC4377021; doi:10.1186/s12864-015-1372-0)

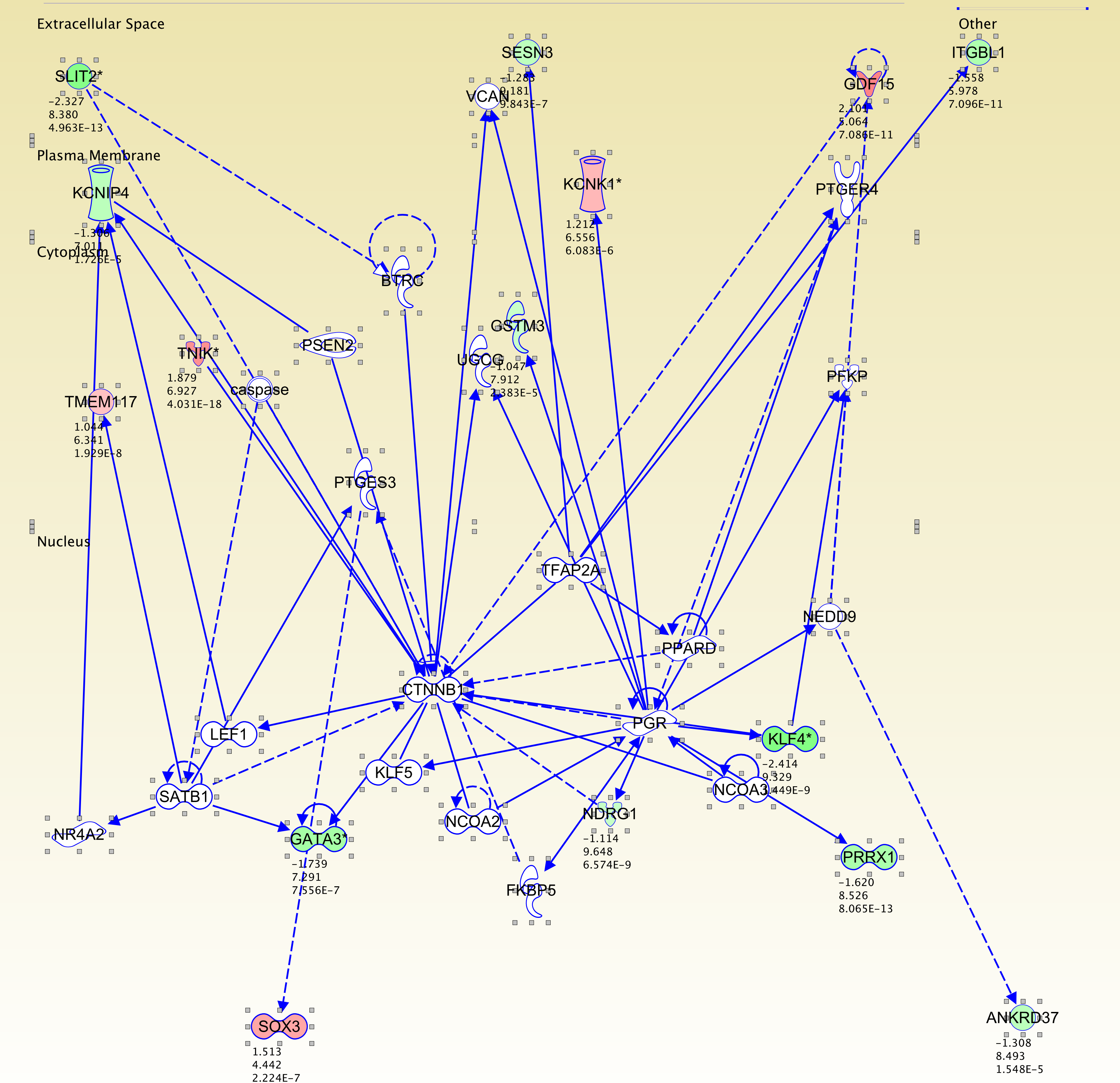

Supplement: Additional file 2 — Figures of the pathways of cell-to-cell signaling as well as cell-death processes, obtained by the DEGs using IPA software. Each figure shows the molecules that were differentially expressed depending on the involved pathway. [file 12864_2015_1372_MOESM2_ESM.zip › CellDeathAndSurvival/FTC/Cell_Death_17_FTC_NT.pdf]

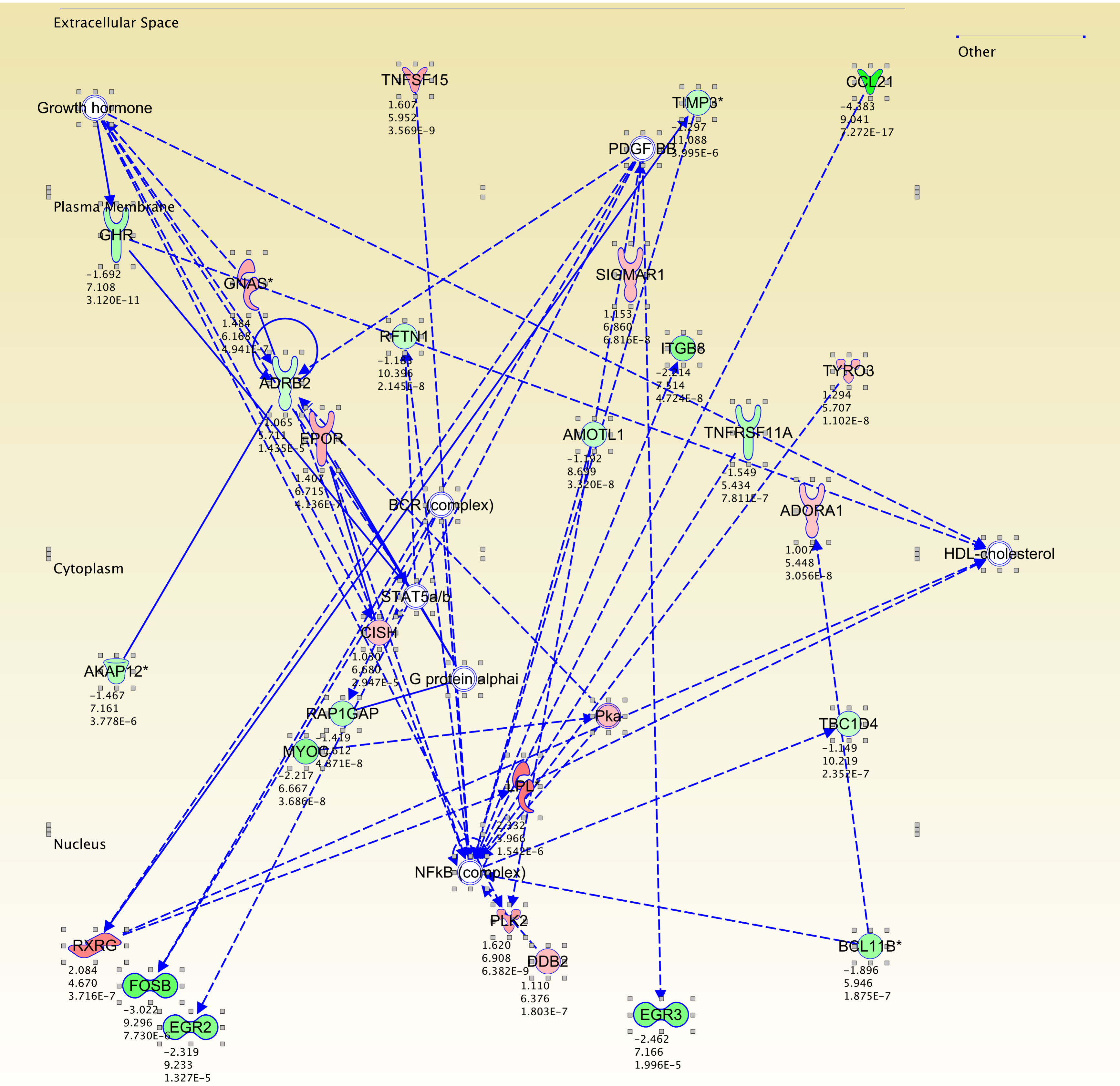

Supplement: Additional file 2 — Figures of the pathways of cell-to-cell signaling as well as cell-death processes, obtained by the DEGs using IPA software. Each figure shows the molecules that were differentially expressed depending on the involved pathway. [file 12864_2015_1372_MOESM2_ESM.zip › CellDeathAndSurvival/FTC/Cell_Death_1_FTC_NT.pdf]

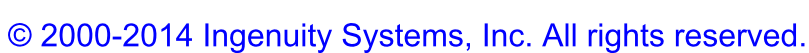

Supplement: Additional file 2 — Figures of the pathways of cell-to-cell signaling as well as cell-death processes, obtained by the DEGs using IPA software. Each figure shows the molecules that were differentially expressed depending on the involved pathway. [file 12864_2015_1372_MOESM2_ESM.zip › CellDeathAndSurvival/FTC/Cell_Death_22_FTC_NT.pdf]

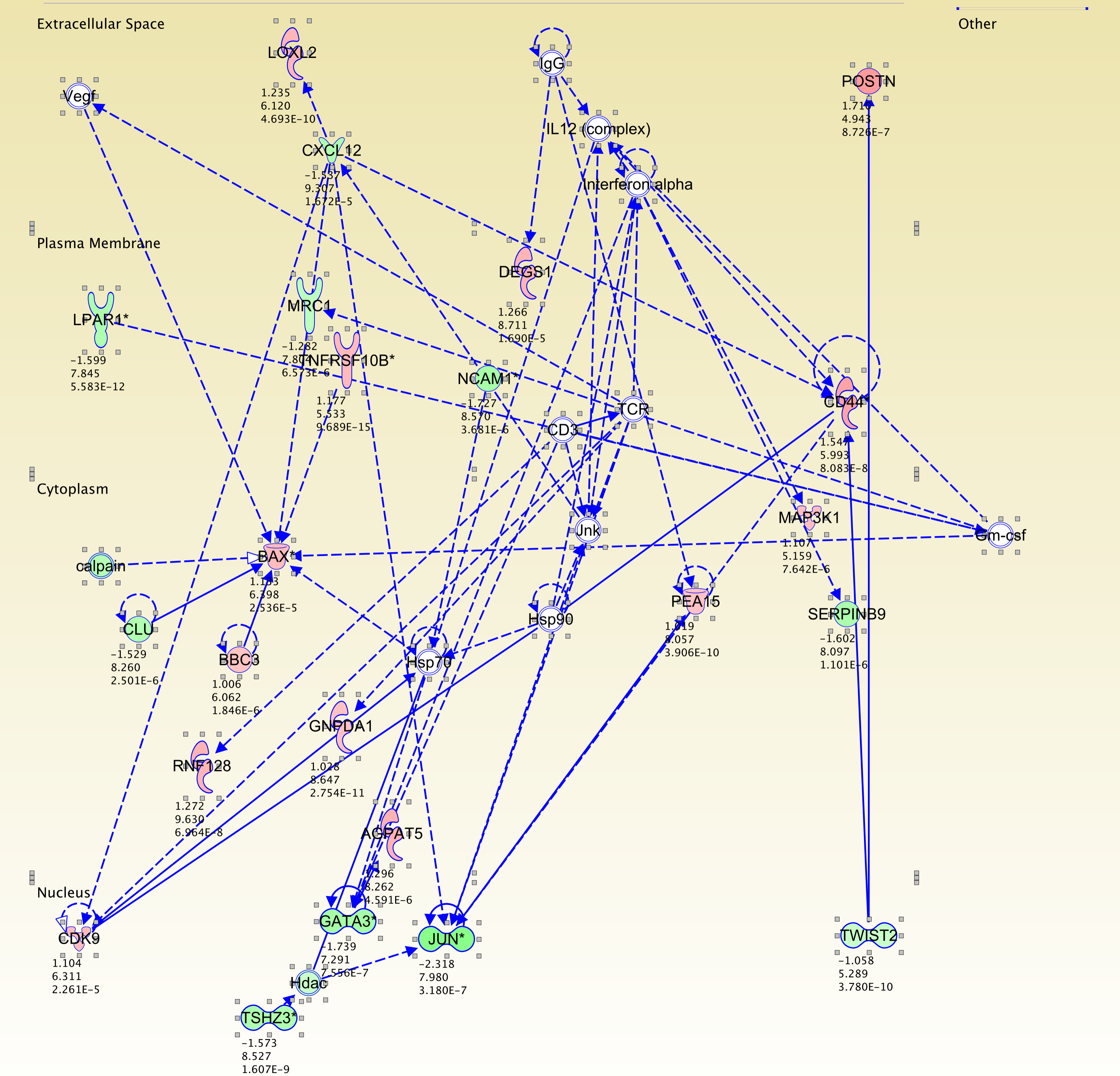

Supplement: Additional file 2 — Figures of the pathways of cell-to-cell signaling as well as cell-death processes, obtained by the DEGs using IPA software. Each figure shows the molecules that were differentially expressed depending on the involved pathway. [file 12864_2015_1372_MOESM2_ESM.zip › CellDeathAndSurvival/FTC/Cell_Death_5_FTC_NT.pdf]

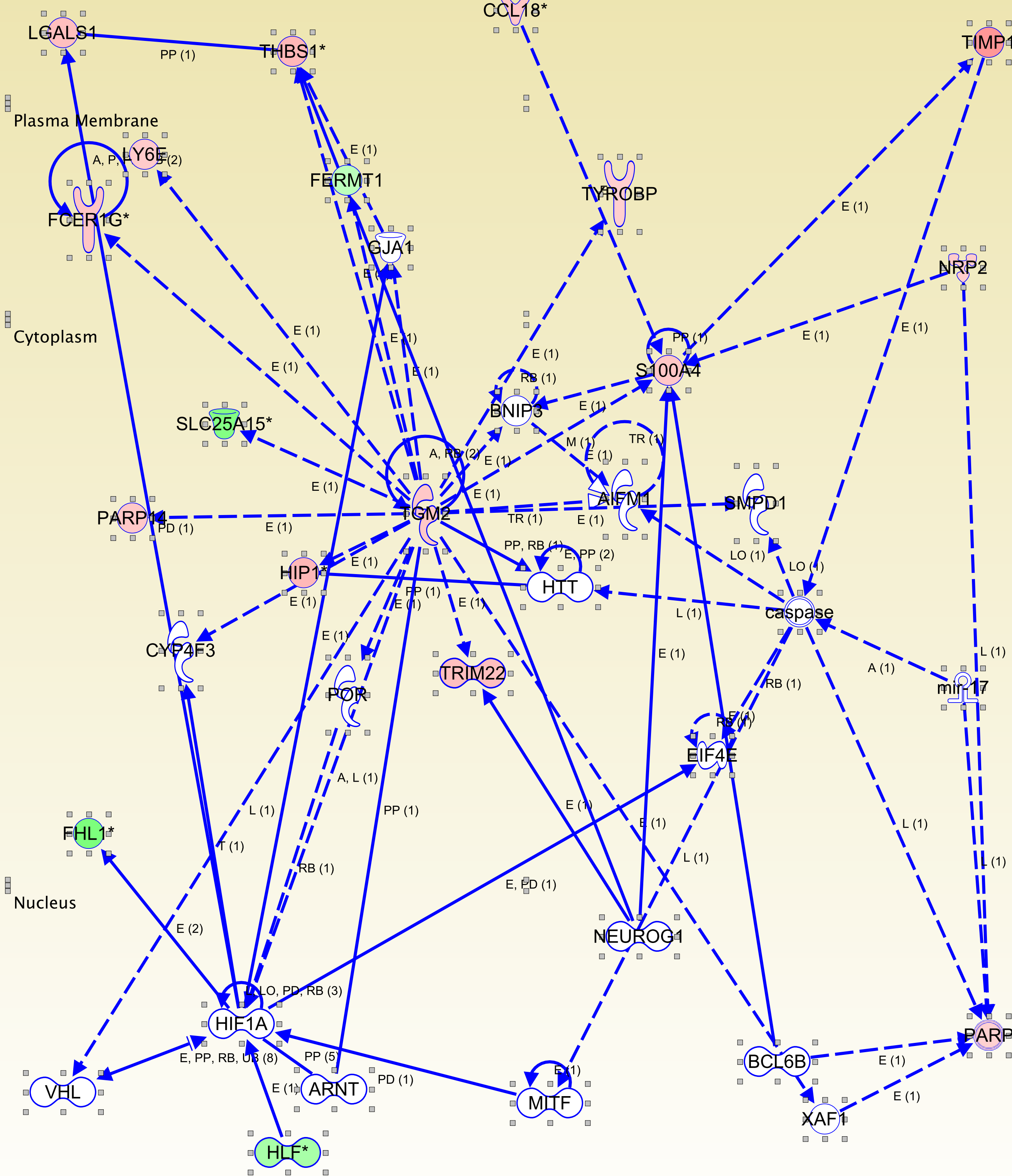

Supplement: Additional file 2 — Figures of the pathways of cell-to-cell signaling as well as cell-death processes, obtained by the DEGs using IPA software. Each figure shows the molecules that were differentially expressed depending on the involved pathway. [file 12864_2015_1372_MOESM2_ESM.zip › CellDeathAndSurvival/PTC/Cell_Death_18_PTC_NT.pdf]

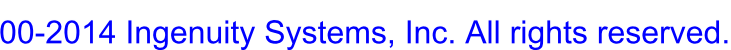

Supplement: Additional file 2 — Figures of the pathways of cell-to-cell signaling as well as cell-death processes, obtained by the DEGs using IPA software. Each figure shows the molecules that were differentially expressed depending on the involved pathway. [file 12864_2015_1372_MOESM2_ESM.zip › CellToCellSignaling/ATC/Cell_Cell_signaling_17_ATC_NT.pdf]

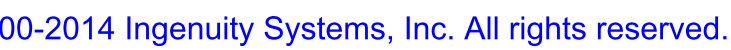

Supplement: Additional file 2 — Figures of the pathways of cell-to-cell signaling as well as cell-death processes, obtained by the DEGs using IPA software. Each figure shows the molecules that were differentially expressed depending on the involved pathway. [file 12864_2015_1372_MOESM2_ESM.zip › CellToCellSignaling/ATC/Cell_Cell_signaling_20_ATC_NT.pdf]

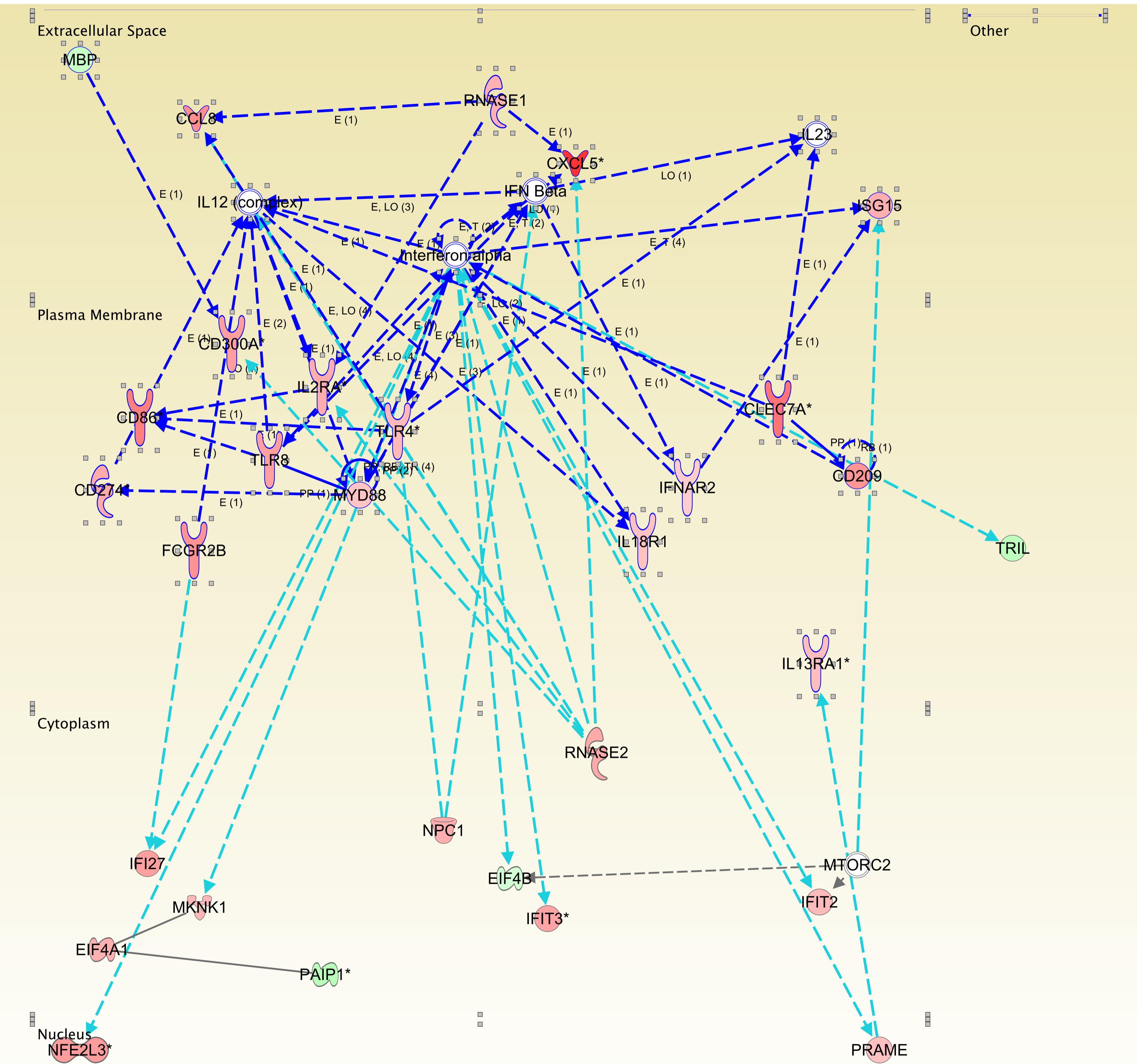

Supplement: Additional file 2 — Figures of the pathways of cell-to-cell signaling as well as cell-death processes, obtained by the DEGs using IPA software. Each figure shows the molecules that were differentially expressed depending on the involved pathway. [file 12864_2015_1372_MOESM2_ESM.zip › CellToCellSignaling/ATC/Cell_Cell_signaling_22_ATC_NT.pdf]

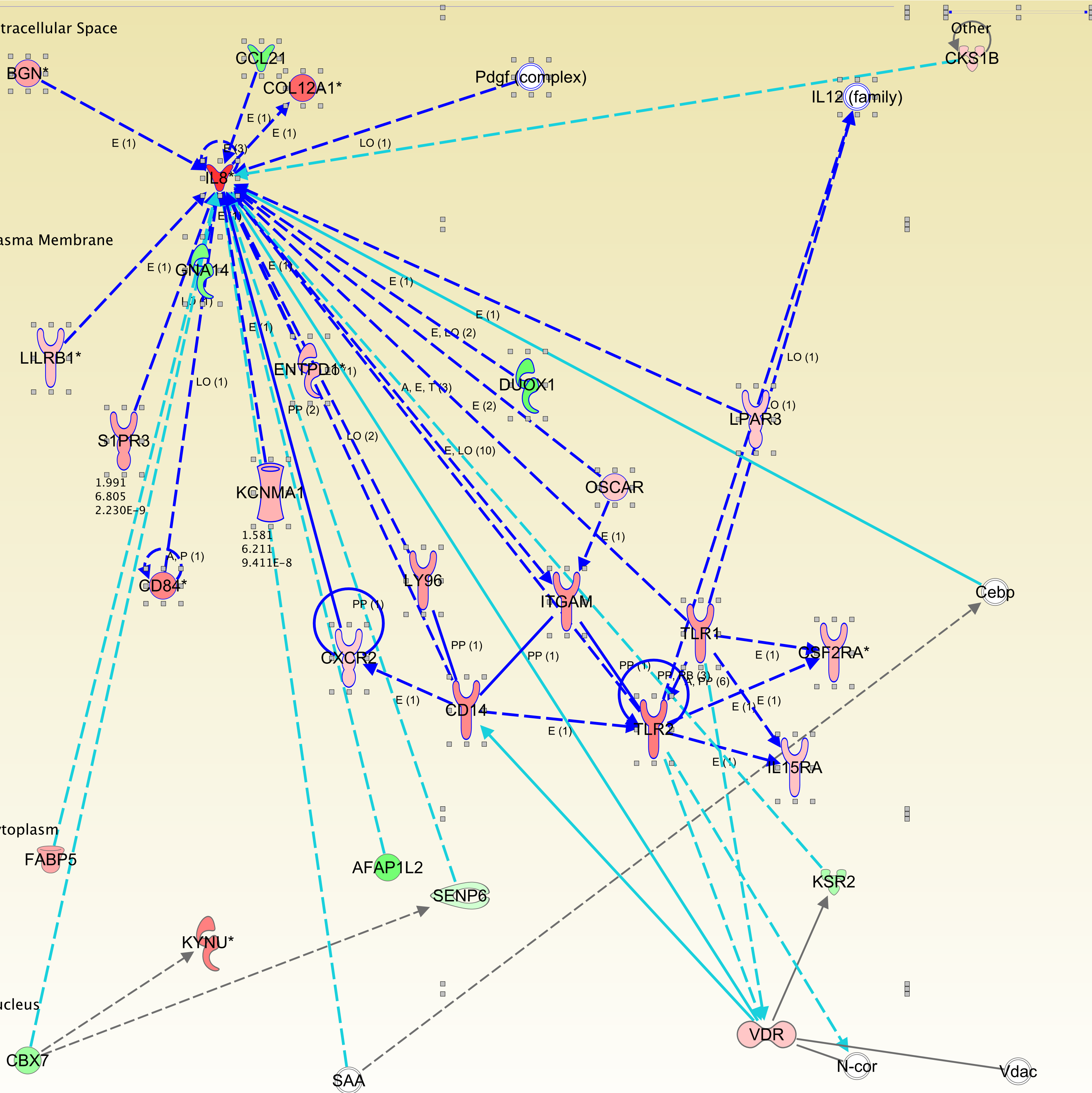

Supplement: Additional file 2 — Figures of the pathways of cell-to-cell signaling as well as cell-death processes, obtained by the DEGs using IPA software. Each figure shows the molecules that were differentially expressed depending on the involved pathway. [file 12864_2015_1372_MOESM2_ESM.zip › CellToCellSignaling/ATC/Cell_Cell_signaling_23_ATC_NT.pdf]

Extracellular Space

Other

Plasma Membrane

Cytoplasm

Nucleus

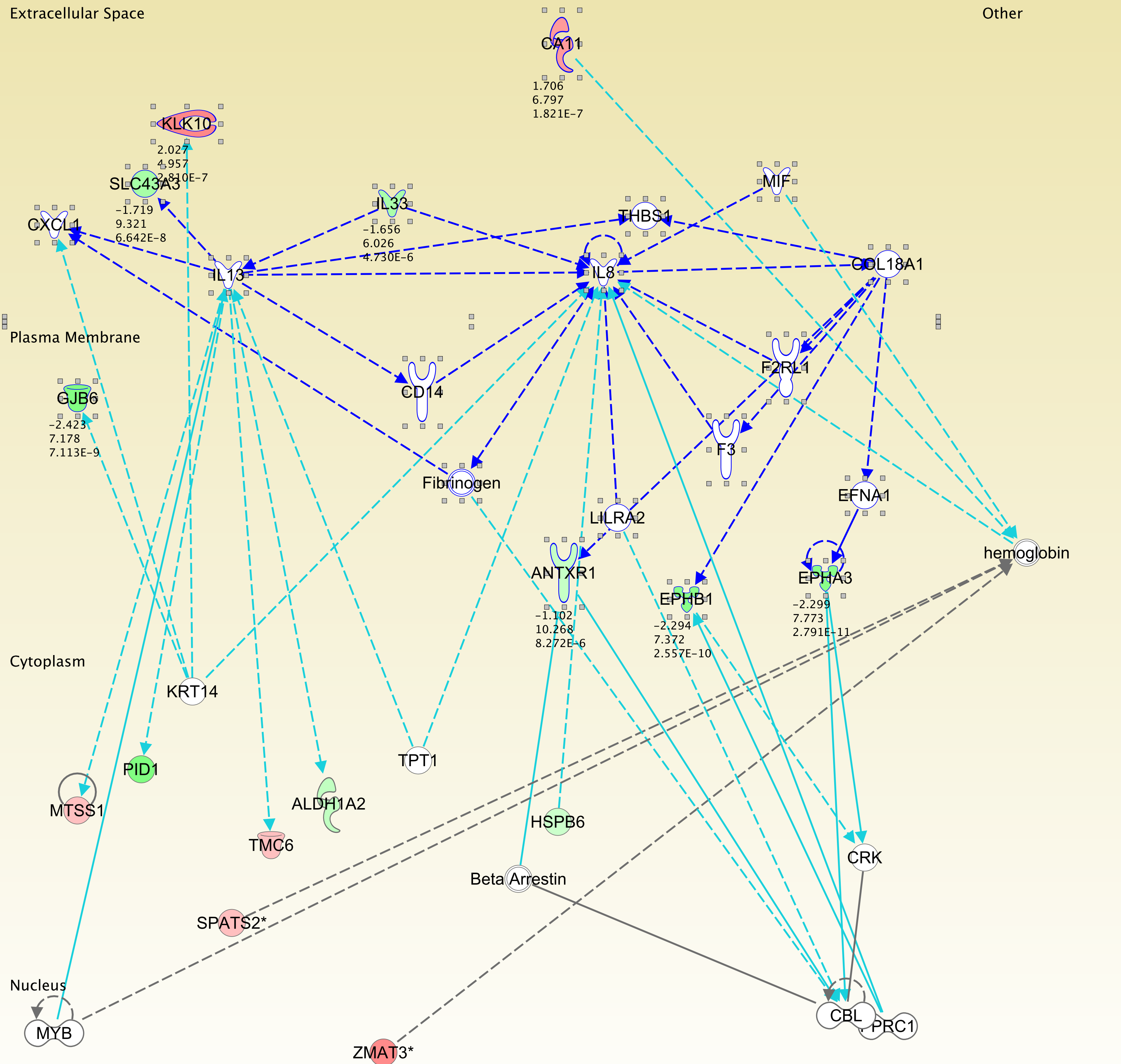

Supplement: Additional file 2 — Figures of the pathways of cell-to-cell signaling as well as cell-death processes, obtained by the DEGs using IPA software. Each figure shows the molecules that were differentially expressed depending on the involved pathway. [file 12864_2015_1372_MOESM2_ESM.zip › CellToCellSignaling/FTC/Cell_Cell_signaling_16_FTC_NT.pdf]

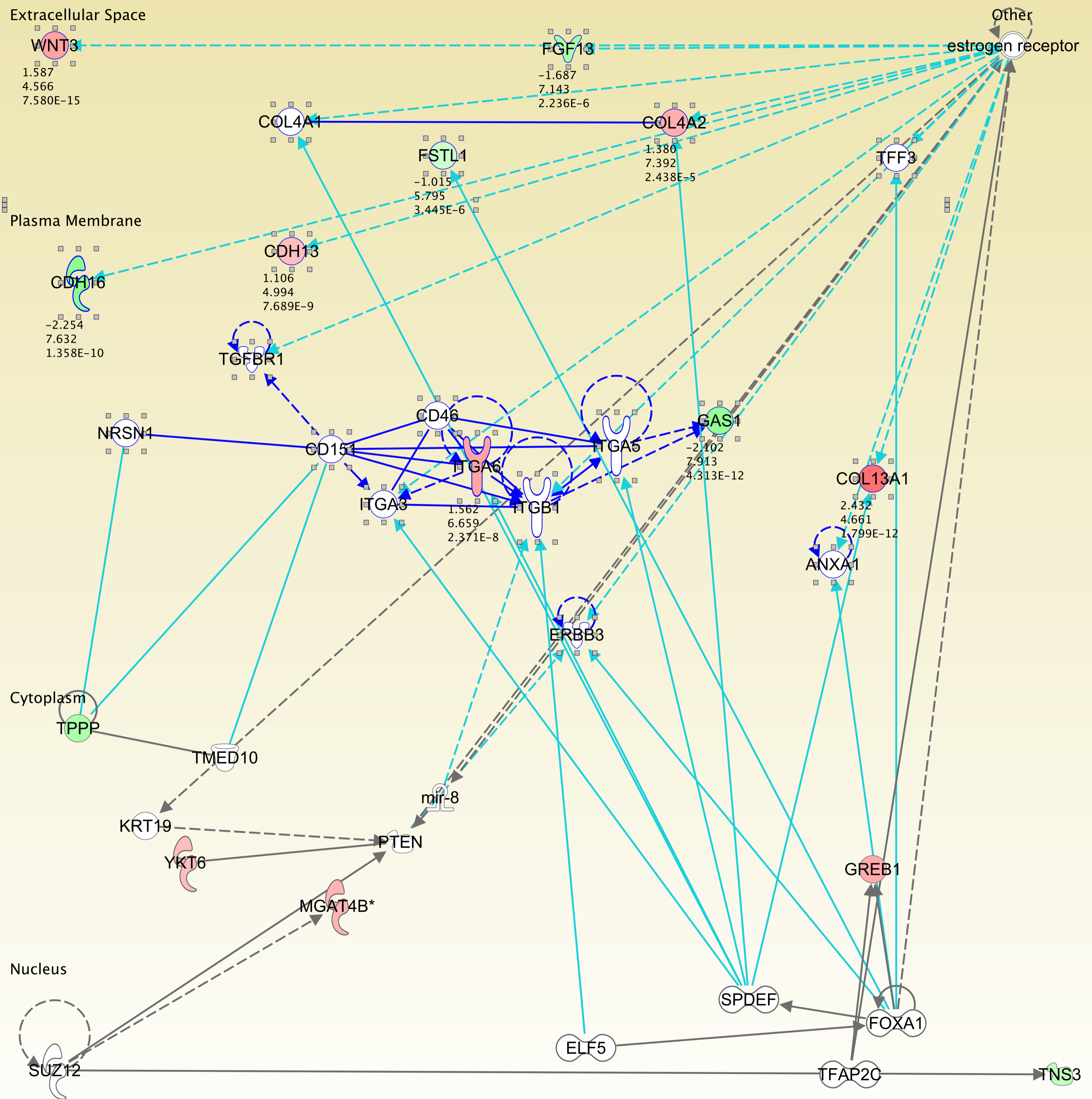

Supplement: Additional file 2 — Figures of the pathways of cell-to-cell signaling as well as cell-death processes, obtained by the DEGs using IPA software. Each figure shows the molecules that were differentially expressed depending on the involved pathway. [file 12864_2015_1372_MOESM2_ESM.zip › CellToCellSignaling/FTC/Cell_Cell_signaling_18_FTC_NT.pdf]

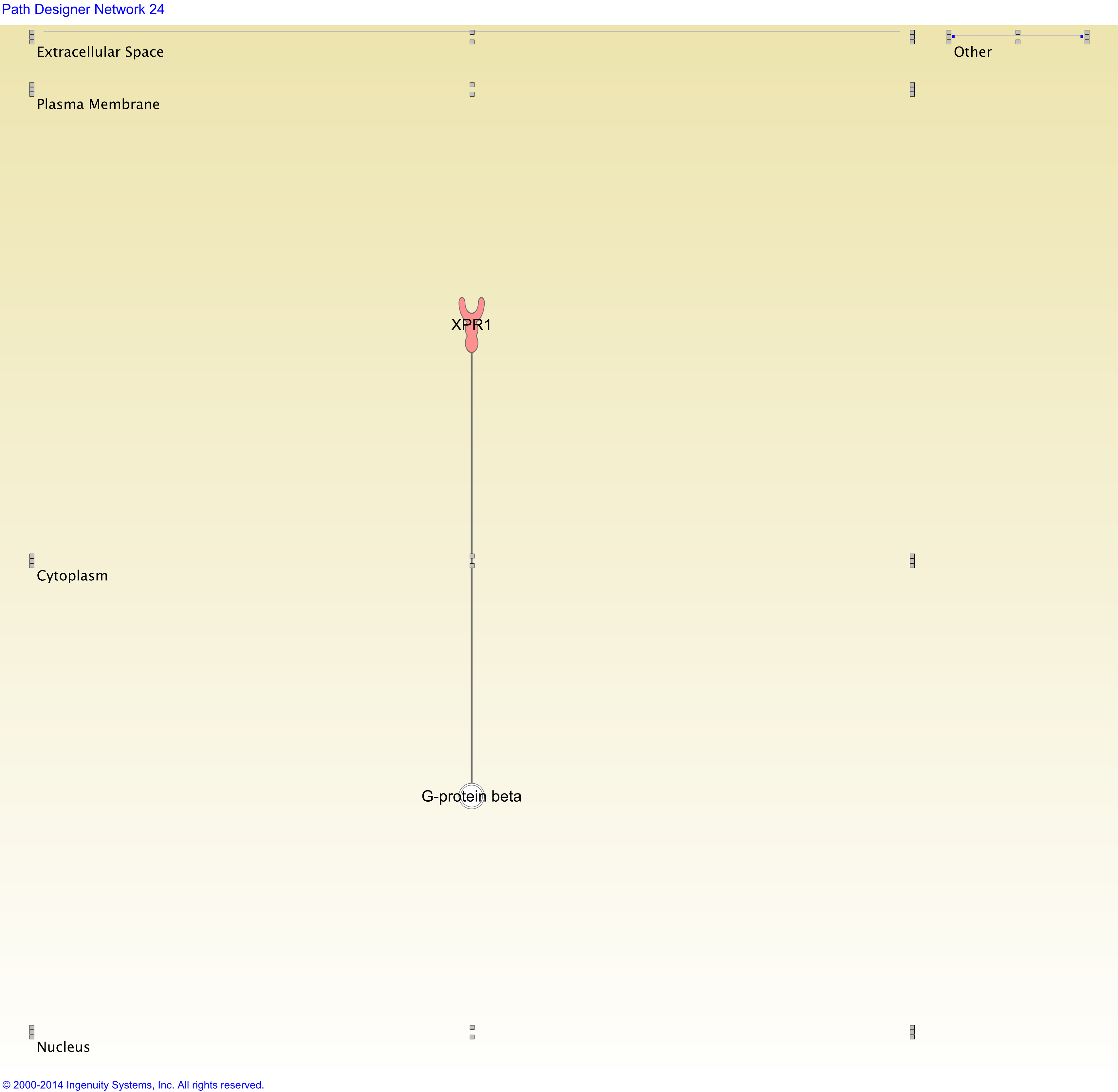

Supplement: Additional file 2 — Figures of the pathways of cell-to-cell signaling as well as cell-death processes, obtained by the DEGs using IPA software. Each figure shows the molecules that were differentially expressed depending on the involved pathway. [file 12864_2015_1372_MOESM2_ESM.zip › CellToCellSignaling/FTC/Cell_Cell_signaling_24_FTC_NT.pdf.pdf]

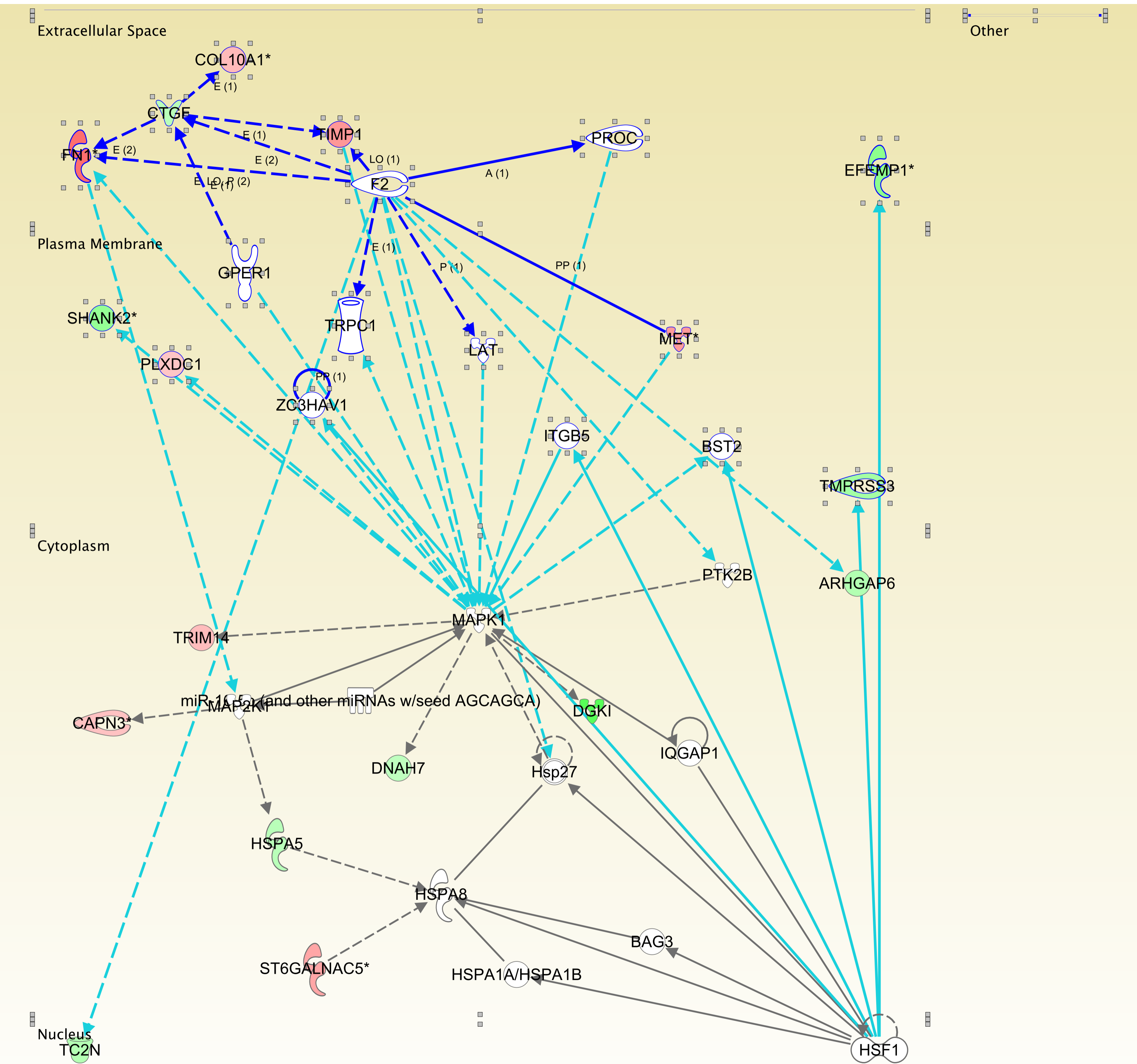

Supplement: Additional file 2 — Figures of the pathways of cell-to-cell signaling as well as cell-death processes, obtained by the DEGs using IPA software. Each figure shows the molecules that were differentially expressed depending on the involved pathway. [file 12864_2015_1372_MOESM2_ESM.zip › CellToCellSignaling/PTC/Cell_Cell_signaling_21_PTC_NT.pdf]

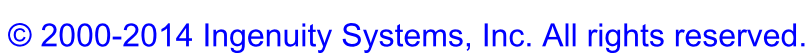

Supplement: Additional file 2 — Figures of the pathways of cell-to-cell signaling as well as cell-death processes, obtained by the DEGs using IPA software. Each figure shows the molecules that were differentially expressed depending on the involved pathway. [file 12864_2015_1372_MOESM2_ESM.zip › CellToCellSignaling/PTC/Cell_Cell_signaling_22B_PTC_NT.pdf]

Nucleus

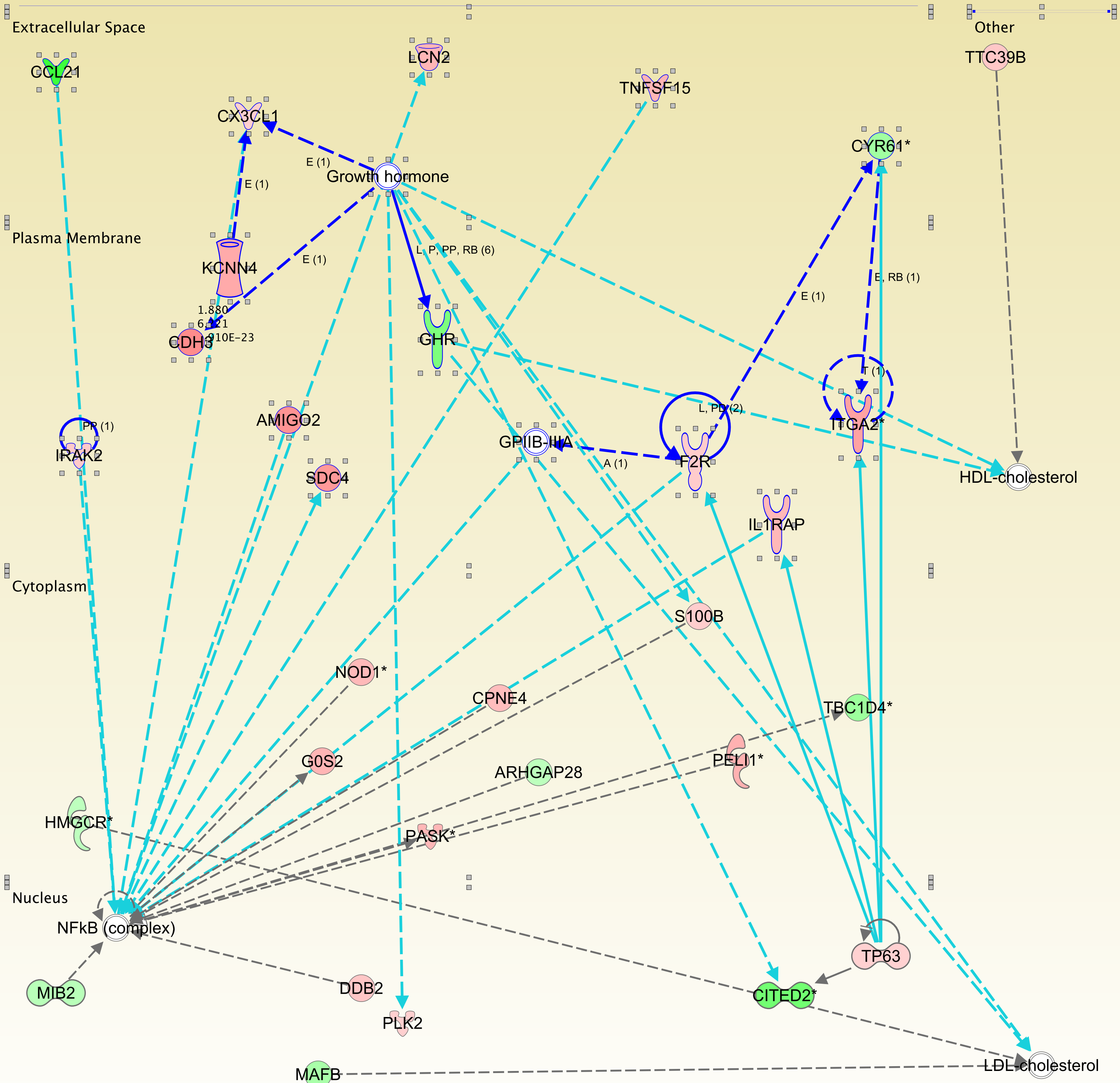

Supplement: Additional file 2 — Figures of the pathways of cell-to-cell signaling as well as cell-death processes, obtained by the DEGs using IPA software. Each figure shows the molecules that were differentially expressed depending on the involved pathway. [file 12864_2015_1372_MOESM2_ESM.zip › CellToCellSignaling/PTC/Cell_Cell_signaling_3_PTC_NT.pdf]

# ATC-NT

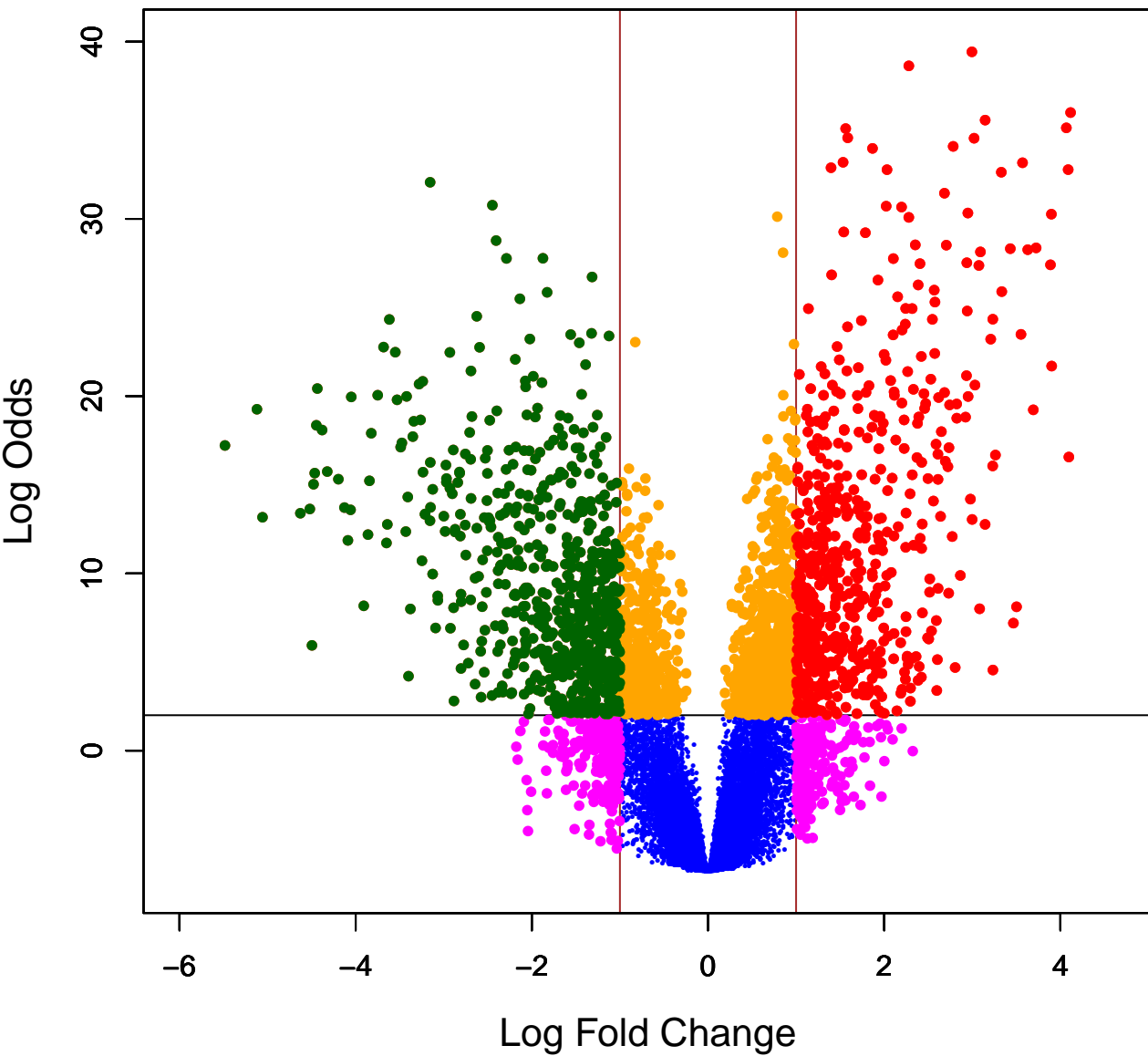

Supplement: Additional file 4 — Validation files. A compressed folder that includes two subfolders (named ValidationSet1 and ValidationSet2, respectively). Those folders include the following files:.csv files with the results of the gene expression differential analysis for the three contrasts (ATC vs normal thyroid tissue, FTC vs normal thyroid tissue and PTC vs normal thyroid tissue)..csv files with the normalized expression matrix for the preprocessing of the validation arrays (each array is identified by its GEO accesion key). PDF files of the volcano plots for the differential expression analysis. [file 12864_2015_1372_MOESM4_ESM.zip › AdditionalFile4/ValidationSet1/volcanoATC-NT.pdf]

# FTC-NT

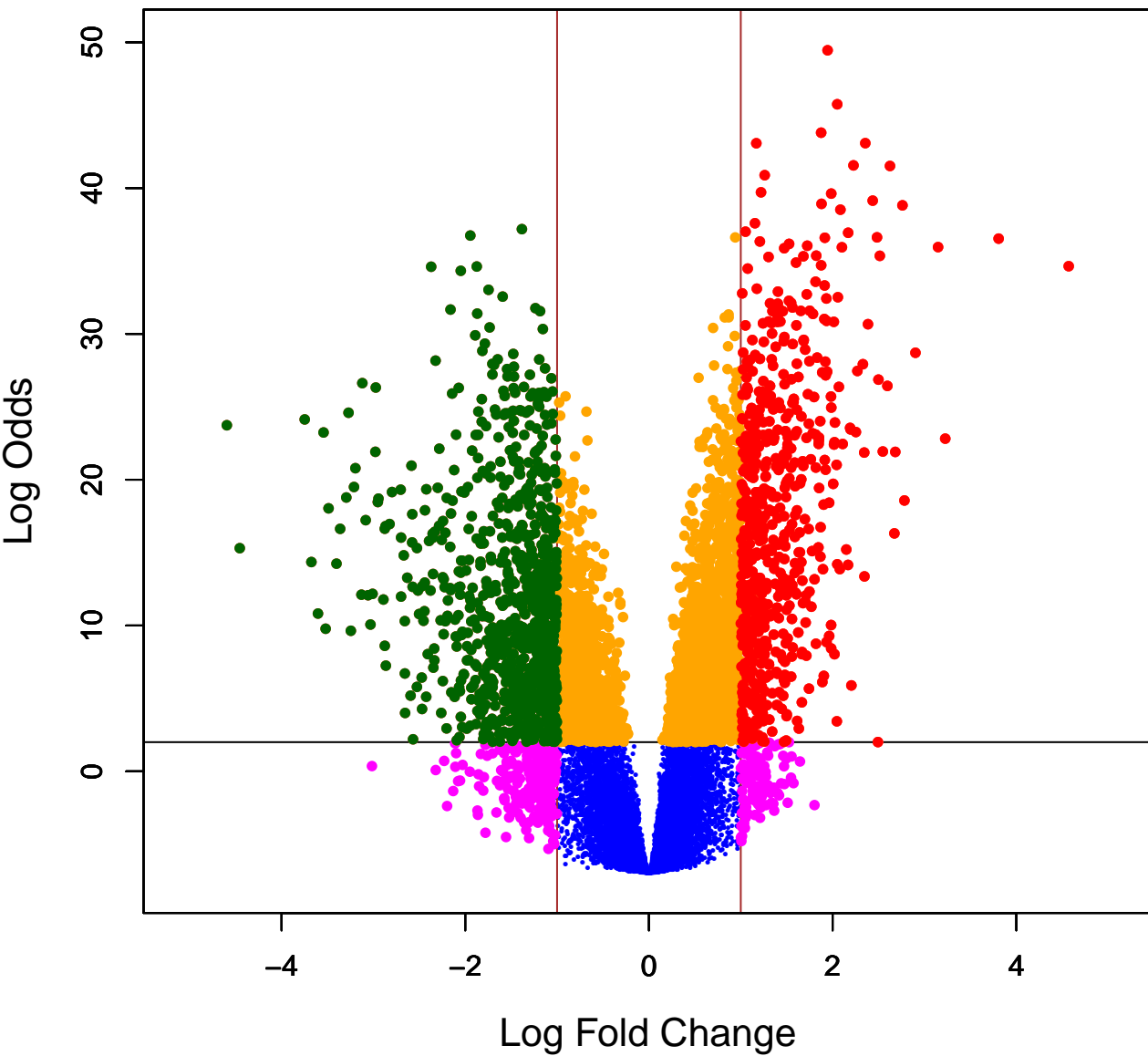

Supplement: Additional file 4 — Validation files. A compressed folder that includes two subfolders (named ValidationSet1 and ValidationSet2, respectively). Those folders include the following files:.csv files with the results of the gene expression differential analysis for the three contrasts (ATC vs normal thyroid tissue, FTC vs normal thyroid tissue and PTC vs normal thyroid tissue)..csv files with the normalized expression matrix for the preprocessing of the validation arrays (each array is identified by its GEO accesion key). PDF files of the volcano plots for the differential expression analysis. [file 12864_2015_1372_MOESM4_ESM.zip › AdditionalFile4/ValidationSet1/volcanoFTC-NT.pdf]

# PTC-NT

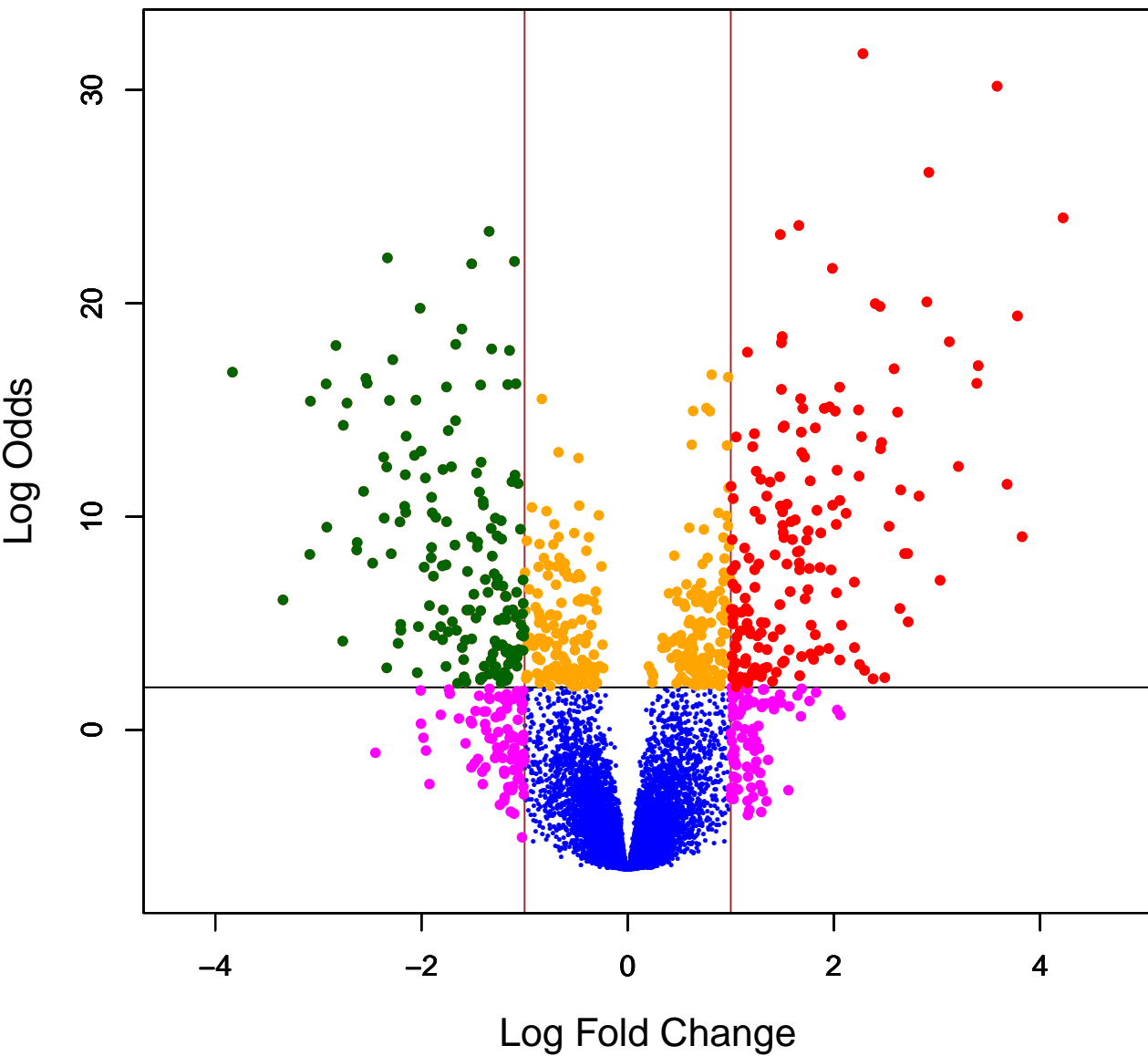

Supplement: Additional file 4 — Validation files. A compressed folder that includes two subfolders (named ValidationSet1 and ValidationSet2, respectively). Those folders include the following files:.csv files with the results of the gene expression differential analysis for the three contrasts (ATC vs normal thyroid tissue, FTC vs normal thyroid tissue and PTC vs normal thyroid tissue)..csv files with the normalized expression matrix for the preprocessing of the validation arrays (each array is identified by its GEO accesion key). PDF files of the volcano plots for the differential expression analysis. [file 12864_2015_1372_MOESM4_ESM.zip › AdditionalFile4/ValidationSet1/volcanoPTC-NT.pdf]

# ATC-Normal

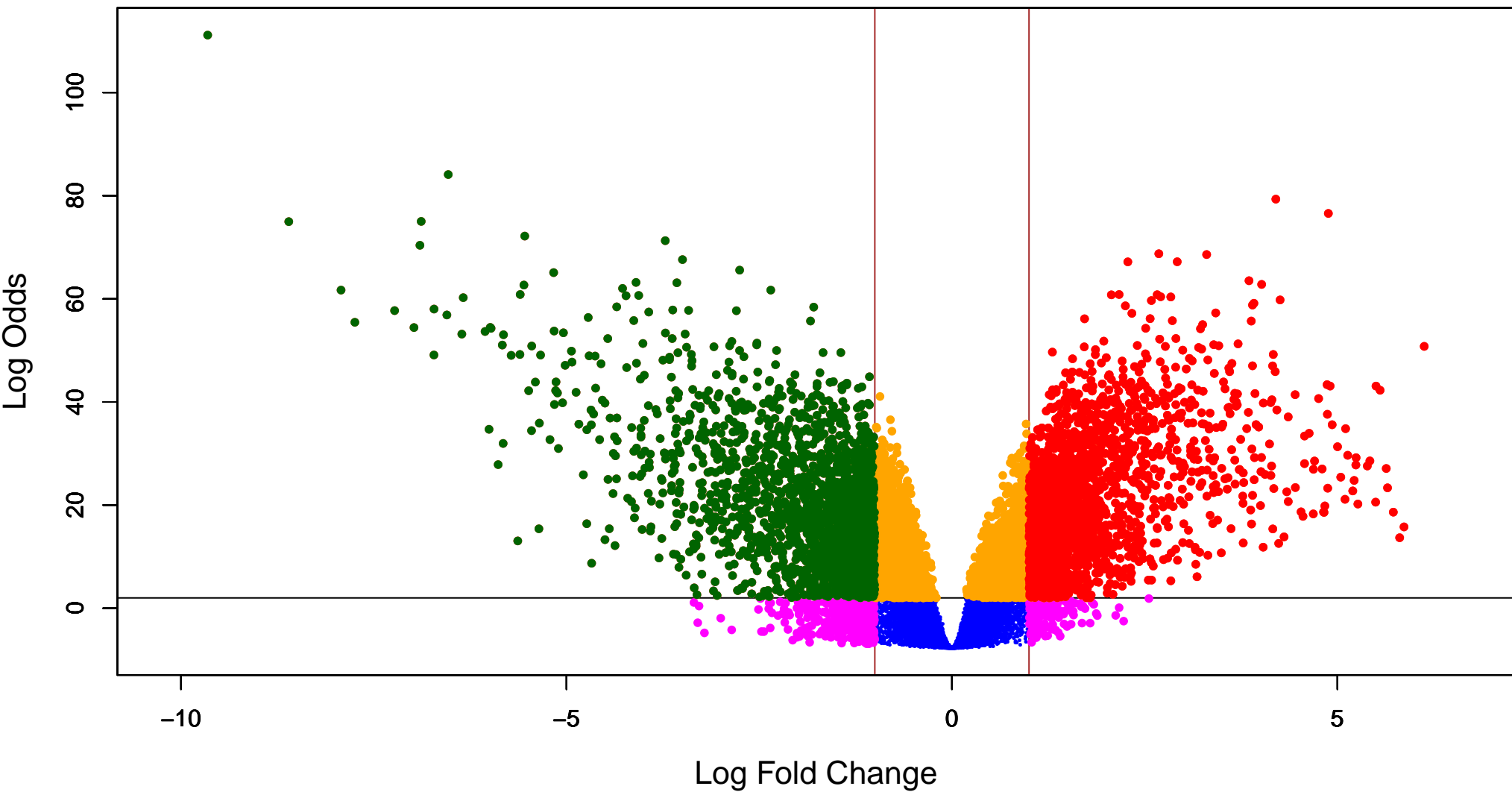

Supplement: Additional file 4 — Validation files. A compressed folder that includes two subfolders (named ValidationSet1 and ValidationSet2, respectively). Those folders include the following files:.csv files with the results of the gene expression differential analysis for the three contrasts (ATC vs normal thyroid tissue, FTC vs normal thyroid tissue and PTC vs normal thyroid tissue)..csv files with the normalized expression matrix for the preprocessing of the validation arrays (each array is identified by its GEO accesion key). PDF files of the volcano plots for the differential expression analysis. [file 12864_2015_1372_MOESM4_ESM.zip › AdditionalFile4/ValidationSet2/VolcanoPlot-ATC-Normal.pdf]

FTC-Normal

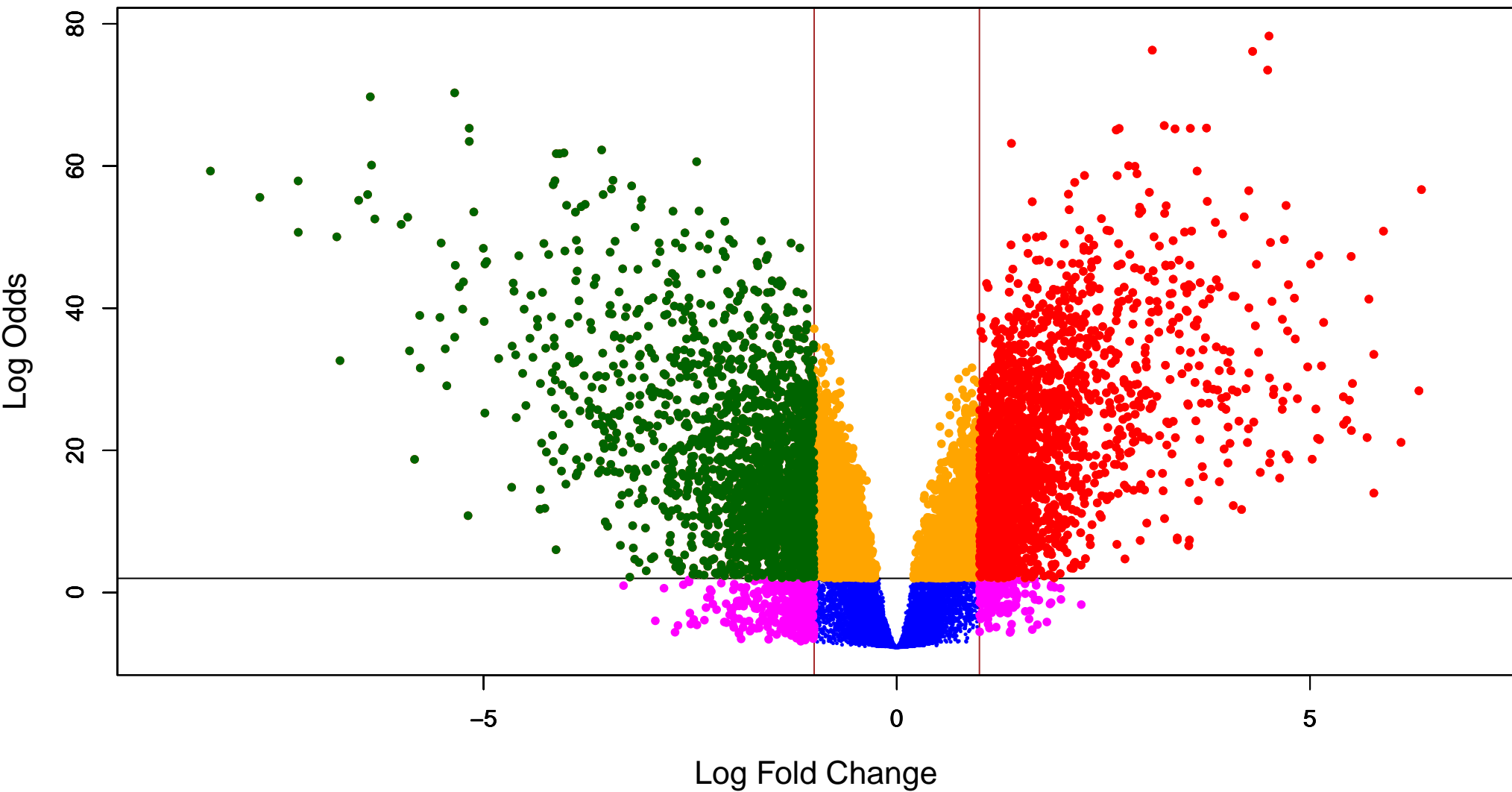

Supplement: Additional file 4 — Validation files. A compressed folder that includes two subfolders (named ValidationSet1 and ValidationSet2, respectively). Those folders include the following files:.csv files with the results of the gene expression differential analysis for the three contrasts (ATC vs normal thyroid tissue, FTC vs normal thyroid tissue and PTC vs normal thyroid tissue)..csv files with the normalized expression matrix for the preprocessing of the validation arrays (each array is identified by its GEO accesion key). PDF files of the volcano plots for the differential expression analysis. [file 12864_2015_1372_MOESM4_ESM.zip › AdditionalFile4/ValidationSet2/VolcanoPlot-FTC-Normal.pdf]

# PTC-Normal

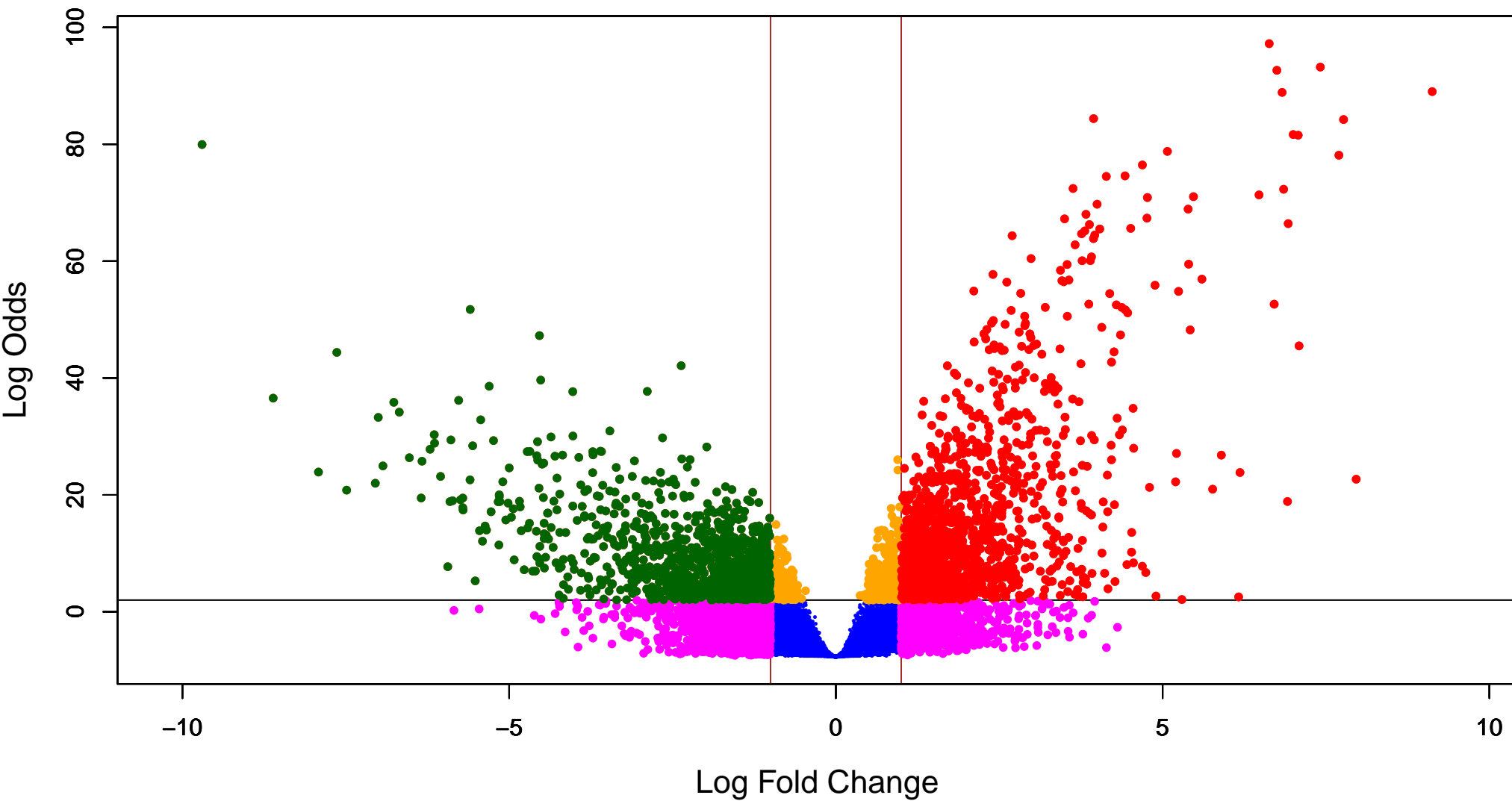

Supplement: Additional file 4 — Validation files. A compressed folder that includes two subfolders (named ValidationSet1 and ValidationSet2, respectively). Those folders include the following files:.csv files with the results of the gene expression differential analysis for the three contrasts (ATC vs normal thyroid tissue, FTC vs normal thyroid tissue and PTC vs normal thyroid tissue)..csv files with the normalized expression matrix for the preprocessing of the validation arrays (each array is identified by its GEO accesion key). PDF files of the volcano plots for the differential expression analysis. [file 12864_2015_1372_MOESM4_ESM.zip › AdditionalFile4/ValidationSet2/VolcanoPlot-PTC-Normal.pdf]
