# Supplementary material for: Genome-wide expression analysis suggests a crucial role of dysregulation of matrix metalloproteinases pathway in undifferentiated thyroid carcinoma
Source: BMC Genomics. 2015 Mar 18;16(1):207. doi: 10.1186/s12864-015-1372-0 (PMC4377021; doi:10.1186/s12864-015-1372-0)
Supplement: Additional file 2 — Figures of the pathways of cell-to-cell signaling as well as cell-death processes, obtained by the DEGs using IPA software. Each figure shows the molecules that were differentially expressed depending on the involved pathway. [file Cell_Death_24_PTC_NT.pdf › CellDeathAndSurvival/PTC/Cell_Death_24_PTC_NT.pdf]

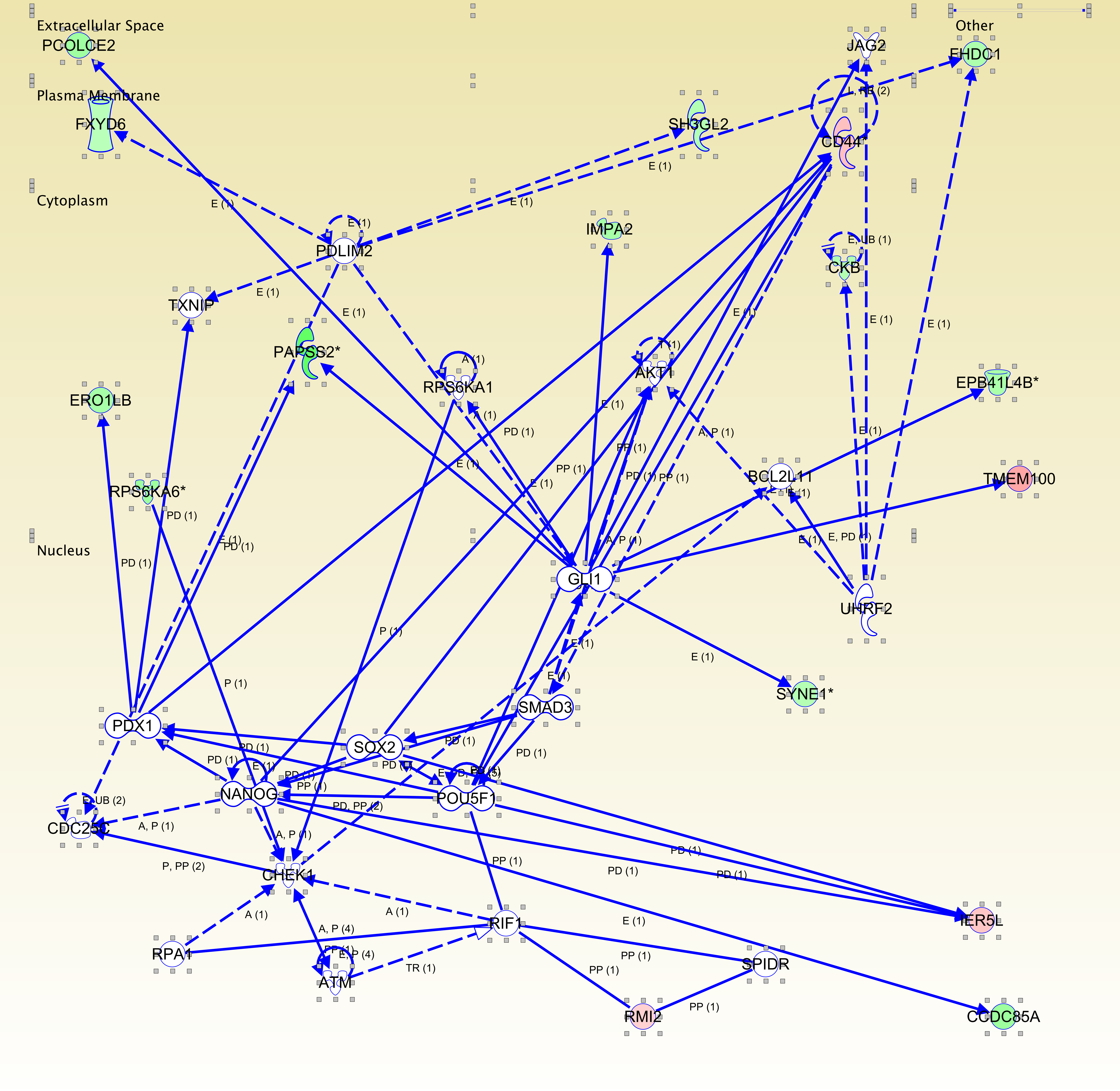

© 2000-2014 Ingenuity Systems, Inc. All rights reserved.
